# Terroir and vintage discrimination of Malbec wines based on phenolic composition across multiple sites in Mendoza, Argentina

**DOI:** 10.1038/s41598-021-82306-0

**Published:** 2021-02-03

**Authors:** Roy Urvieta, Gregory Jones, Fernando Buscema, Rubén Bottini, Ariel Fontana

**Affiliations:** 1 Grupo de Bioquímica Vegetal, Instituto de Biología Agrícola de Mendoza, Facultad de Ciencias Agrarias, CONICET-Universidad Nacional de Cuyo, Almirante Brown 500, Chacras de Coria, M5528AHB Mendoza, Argentina; 2 Catena Institute of Wine, Bodega Catena Zapata, Cobos s/n, Agrelo, M5509 Mendoza, Argentina; 3 Evenstad Center for Wine Education, Linfield University, McMinnville, OR USA; 4 Instituto de Veterinaria Ambiente y Salud, Universidad Juan A. Maza, Lateral Sur del Acceso Este 2245, 5519 Guaymallén, Argentina; 5 Cátedra de Química Orgánica y Biológica, Departamento de Biomatemática y Fisicoquímica, Facultad de Ciencias Agrarias-Universidad Nacional de Cuyo, Chacras de Coria, Mendoza Argentina

**Keywords:** Small molecules, Chemical tools, Climate sciences

## Abstract

This study evaluated the phenolic profiles of Malbec wines made from grapes of 23 parcels distributed in 12 geographical indications (GIs) from Mendoza, Argentina. Wines were elaborated under standardized winemaking conditions over three consecutive vintages (2016–2018). Data discriminated wines from different GIs and parcels, based on an integrative data analysis by chemometric tools. Vintage effect and specific phenolic compounds were associated with some GIs or parcels. As well, regional climate conditions allowed partial discrimination of the GIs (and also some parcels). A random forest analysis correctly identified 11 out of 23 individual parcels across the different vintages. The most notorious compounds associated with such classification were *p*-coumaric acid, delphinidin-3-*O*-glucoside, caffeic acid, quercetin and peonidin-3-*O*-glucoside. The presented research allows to individualize, through phenolic profiles, parcels with unique characteristics over years. This is the first report characterizing Malbec wines coming from several GIs (and individual parcels) in different vintages. These results are strongly related to terroir features of wines, contributing to a better communication to consumers and to position Argentinean wines.

## Introduction

Wine is composed by a complex scenery of chemistry-related species that come out from an interplay amongst environmental, genetic and human factors^1^. Grapevine variety, site, year (vintage), and quality ratings—are features used for the characterization and description of wines which are a reflection of the structure and quantity of small molecules^2^. In viticulture the concept of “terroir” refers to this interplay of factors, which include the vine and its physical environment, together with the human managing in the vineyards and wineries^3^. Therefore, local climate and soil features, along with grape varieties, is at the core of terroir^4^. However, terroir remains one of the most intriguing challenges in today wine world, largely because what terroir encompasses is not universally understood or accepted^4^. Nevertheless, an increasing number of scientists are committed to identify help identify the important aspects of terroir that define the boundaries between nature and nurture^5^. As such, the study of terroir has developed over the last 20 years following five main areas of study: (1) terroir components that influences vine growth through examination of the climate–soil–water relationships; (2) terroir components that influences fruit composition and wine quality; (3) regional fingerprinting of wines (chemical signatures); (4) viticulture zoning (finding the best terroirs); and (5) precision viticulture (spatial technologies to manage and improve the crop). Historically, the term commonly refers to a rather small area whose soil and microclimate impart distinctive qualities to the fruit and wines. Recent research has shown that metabolites in wine are affected by the terroir^6^. As well, the place of each vineyard represents a single terroir, which is displayed in the obtained wines that are more or less consistent amongst years^6^. This definition of an exclusive place giving a unique wine is exploited by different winemaking regions worldwide to differentiate their terroirs, revealing distinguishing characteristics that add economic value. In France, where the concept of terroir was born, the reputation of certain regions such as Bordeaux or Burgundy as producers of exclusive wines transcends local boundaries. In fact, such terroirs position the country in the minds of international consumers as a producer of high-quality wines.

Extensive research has detailed the strong effect that climatic conditions of each vintage and regional variations in soil types have on the berry and wines composition^1,6–9^. Most of these studies agree that vintage variations have the most significant impact on composition. However, some important terroir differences may be achieved by analysing individual characteristics of each terroir (or site) on wines from a given vintage. The possibility to identify any consistent behaviour for specific parcels or terroirs over different vintages appears as a challenging task to validate the concept of exclusive, terroir-driven wines.

**Figure 1:**
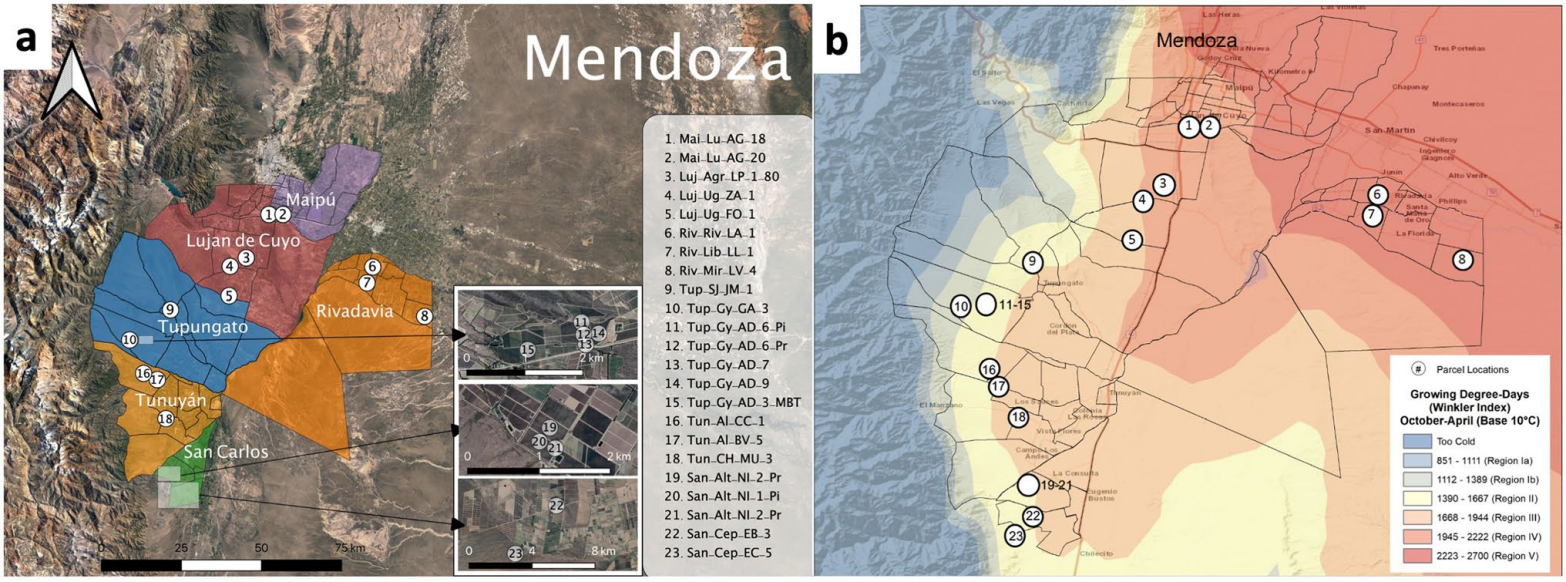
(**a**) Distribution of parcels in 12 GIs belonging to six Departments of the three main viticulture zones, First Zone, East Zone and Uco Valley. (**b**) Average growing degree-days (GDD), over Mendoza derived from TerraClimate 1958–2019 with a monthly temporal resolution and a ~ 4-km (1/24th degree) spatial resolution^14^. GDD class limits originally given by Amerine and Winkler^15^ along with lower and upper bounds for Region I and Region V (see text). The images were created PowerPoint, QGIS.org, 2021. QGIS Geographic Information System. QGIS Association. http://www.qgis.org and ArcGIS 10.8; www.esri.com.

Malbec (*Vitis vinifera* L. cv. Malbec) is a red grape variety that was originated in France. It is also known there as Côt Noir or Auxerrois, and it is still grown in the Cahors and Bordeaux regions. Today, Argentina has the highest acreage of Malbec vineyard, representing approximately 77% of the world production, followed by France, Chile, and the United States^10^. The Malbec variety is emblematic for Argentina’s winemaking industry, being the most cultivated in the country with an area of 43,000 ha. Eighty five of the total production is located in the province of Mendoza^11^. The majority of Argentina’s most renowned Malbec wines come from Mendoza’s high elevation regions such as Uco Valley and Luján de Cuyo, located at the foothills of the Andes Mountains at altitudes between 900 and 1600 m a.s.l. (Fig. 1). These high elevated regions have semi-arid to arid climate, with usually less than 300 mm of annual precipitation. In addition, the majority of the annual precipitation occurs during the growing season from October to April^12^. Average annual temperature is approximately 17 °C with only slight risk of extreme cold or hot temperatures. During the growing season temperatures roughly average20 °C, with high levels of solar radiation^12^. These conditions are quite optimal for Malbec grapes which typically require an intermediate to warm climate, with high solar potential, and low rainfall (along with specific vineyard management techniques)^13^.

Phenolic compounds (PCs) play important roles in the quality perception of red wines, contributing to their mouthfeel, colour and potential aging^16,17^. The PCs are mainly anthocyanins, flavan-3-ols, flavonols, phenolic acids (including hydroxybenzoic acids and hydroxycinnamic acids) and stilbenes^16^. The majority of these compounds originate from the grapes themselves, while some others are generated through chemical and biochemical reactions during fermentation and aging. As a result, the profile and concentration of PCs in wines are affected by the grape variety, soil characteristics, climatic conditions, vintage effect, vineyard management, and fermentation and aging conditions^18^. In the case of fermentation and aging, modification in PCs structure are mainly related to oxidative reactions, and chain reactions with oxidation products^19,20^. If a single variety of a non-aged wine is considered, the composition and content of PCs mostly depends on the origin of grapes and vintage. In this sense, PCs have been proposed as chemical markers to establish variety authenticity, vintage effect and geographical origin from various regions of the world^1,6,9,21–24^, . For Malbec, in a recent research we performed a comprehensive study of wines from different locations of Mendoza by evaluating the phenolic and sensory profiles with their possible associations to a single vintage^25,26^. By using chemical data, a clearer separation of wines from different locations was achieved as compared to using the sensory data alone. However, the previous study only examined one vintage and the geographical differentiation was only achieved for relatively large regions. Taking into economic potential that the differentiation of specific sites has for the Argentinean wine industry, we propose to integrate data over three vintages to help classify Malbec wines from different GIs and unique parcels inside those GIs.

Therefore, the purpose of this study was to examine Malbec wines coming from 23 parcels distributed across 12 GIs from six Departments of Mendoza, Argentina (Fig. 1 and Table 1). Wines from these parcels were made under standardized winemaking conditions over three consecutive vintages (2016, 2017, 2018) and analysed for their phenolic profiles. The various parcels where grapes were obtained included different environmental (climate and soil) conditions. They represented the three most important regions of Malbec wine production of Argentina in terms of quantity and quality, including the First Zone, the East Zone and the Uco Valley (Fig. 1). Different chemometric tools were used for (1) to classify wines according to vintage, (2) to associate zones, departments and individual GIs with their phenolic composition, and (3) to assess the potential to identify individual parcels over different vintages.

**Table 1:** Mendoza Malbec parcels information.

Locations								Harvest date			
Zones	Departments	GIs	Parcels	Planting year	Vineyard Orintation	Latitude	Longitude	Elevation (m)	2016	2017	2018
First Zone	Lujan de Cuyo	Agrelo	Luj-Agr-LP-1–80	2006	N-S	33° 9′58.02″S	68°54′53.35″W	959	5-abr	6-mar	12-mar
		Ugarteche	Luj-Ug-FO-1	2008	N-S	33°16′11.98″S	68°58′28.41″W	1051	17-mar	16-mar	26-mar
			Luj-Ug-ZA-1	2001	N-S	33°11′38.22″S	68°57′21.67″W	981	17-mar	31-mar	28-mar
	Maipu	Lunlunta	Mai-Lu-AG-18	1922	N-S	33° 2′58.31″S	68°50′54.22″W	928	23-mar	14-mar	20-mar
			Mai-Lu-AG-20	1922	N-S	33° 3′6.35″S	68°50′38.90″W	929	23-mar	15-mar	21-mar
East Zone	Rivadavia	El Mirador	Riv-Mir-LV-4	2001	*	33°18′30.24″S	68°19′25.15″W	635	18-mar	8-mar	21-mar
		La Libertad	Riv-Lib-LL-1	1921	N-S	33°13′15.03″S	68°30′10.64″W	671	28-mar	14-mar	22-mar
		Rivadavia	Riv-Riv-LA-1	2003	N-S	33°11′19.88″S	68°29′48.59″W	671	22-mar	10-mar	22-mar
Uco Valley	San Carlos	Altamira	San-Alt-NI-1-Pi	2000	N-S	33°45′22.92″S	69°10′40.91″W	1100	6-abr	22-mar	12-mar
			San-Alt-NI-1-Pr	2000	N-S	33°45′25.96″S	69°10′34.54″W	1100	7-abr	23-mar	12-mar
			San-Alt-NI-2-Pr	2000	N-S	33°45′20.10″S	69°10′38.89″W	1100	7-abr	24-mar	14-mar
		El Cepillo	San-Cep-EB-3	2005	N-S	33°48′39.39″S	69° 10′7.95″W	1079	28-mar	17-mar	16-mar
			San-Cep-EC-5	2010	N-S	33°50′21.35″S	69°11′44.80″W	1104	31-mar	20-mar	12-mar
	Tunuyan	Chacayes	Tun-Ch-MU-3	2005	N-S	33°36′46.67″S	69°11′39.55″W	1006	13-abr	22-mar	29-mar
		Los Arboles	Tun-Al-BV-5	2005	N-S	33°32′37.22″S	69°14′29.37″W	1130	11-abr	22-mar	22-mar
			Tun-Al-CC-1	2002	N-S	33°32′16.33″S	69°14′45.98″W	1138	11-abr	22-mar	22-mar
	Tupungato	Gualtallary	Tup-Gy-AD-3-MBT	1998	N-S	33°23′56.47″S	69°15′34.29″W	1384	21-abr	27-mar	23-mar
			Tup-Gy-AD-6-Pi	1999	N-S	33°23′37.26″S	69°14′59.06″W	1349	15-abr	13-mar	14-mar
			Tup-Gy-AD-6-Pr	1999	N-S	33°23′42.29″S	69°14′58.62″W	1350	13-abr	28-mar	22-mar
			Tup-Gy-AD-7	1999	N-S	33°23′48.65″S	69°14′57.03″W	1346	6-abr	24-mar	13-mar
			Tup-Gy-AD-9	1999	N-S	33°23′43.28″S	69°14′50.31″W	1340	15-abr	24-mar	22-mar
			Tup-Gy-GA-3	2011	N-S	33°24′3.40″S	69°18′7.25″W	1510	20-abr	3-abr	28-mar
		San José	Tup-SJ-JM-1	2007	N-S	33°18′59.23″S	69°10′5.37″W	1240	15-abr	22-mar	27-mar

## Results and discussion

A total of 23 Malbec single-parcel wines, produced under standardized winemaking conditions during three vintages (2016, 2017 and 2018), were studied. Wine samples belonged to three Zones (large regions), including six Departments (political divisions) of Mendoza, Argentina and corresponding to 12 GIs (Fig. 1 and Table 1). Each sample was evaluated for PCs ((anthocyanins and low molecular weight (LMW)-PCs)) using high-performance liquid chromatography with diode array detection (HPLC–DAD). Dataset included 27 compounds quantified and used to classify and/or to discriminate Argentinean Malbec wines. They included 12 anthocyanins and 15 LMW-PCs comprising phenolic acids, flavanols, flavonols, stilbenes and phenyl-ethanol analogues. Supplementary Tables S1-S4 compiles the overall data that were used to perform the statistical analysis.

### Vintage effect

**Figure 2:**
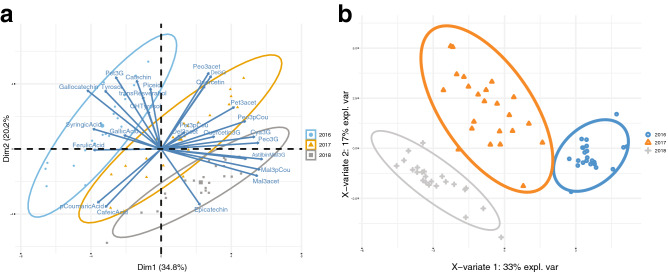
(**a**) Principal component analysis biplot of combined PCs measured in individual fermentation replicates of Malbec wines from the 2016, 2017 and 2018 vintages. Ellipses that overlap are not significantly different from one another at the 95% level. (**b**) PLS-DA plot for the vintages. Samples of 2016 are represented with color blue, 2017 with orange, and 2018 with grey. Legend: Del3G = Delphinidin 3-*O*-glucoside, Cya3G = Cyanidin 3-*O*-glucoside, Pet3G = Petunidin 3-*O*-glucoside, Peo3G = Peonidin 3-*O*-glucoside, Mal3G = Malvidin 3-*O*-glucoside, Del3acet = Delphinidin 3-*O*-acetylglucoside, Pet3acet = Petunidin 3-*O*-acetylglucoside, Peo3acet = Peonidin 3-*O*-acetylglucoside, Mal3Acet = Malvidin 3-*O*-acetylglucoside, Pet3pCou = Petunidin 3-*O*-*p*-coumaroylglucoside, Peo3pCou = Peonidin 3-*O*-p-coumaroylglucoside, Mal3pCou = Malvidin 3-*O*-*p*-coumaroylglucoside, GallicAcid = Gallic acid, OHTyrosol = OH-tyrosol, Tyrosol = Tyrosol, Catechin = (+)-Catechin, SyringicAcid = Syringic acid, Epicatechin = (−)-Epicatechin, Astilbin = Astilbin, CafeicAcid = Caffeic acid, pCoumaricAcid = *p*-coumaric acid, FerulicAcid = Ferulic acid, Piceid = *trans*-piceid, transResveratrol = *Trans*-resveratrol, Quercetin3G = Quercetin-3-glucoside, Quercetin = Quercetin, Gallocatechin = (−)-Gallocatechin. The figure was generated using Adobe Illustrator, version 22.1.0 (https://www.adobe.com/products/illustrator.html), Rpackage factoextra—‘factoextra’ and R-package mixOmics—‘mixOmics’.

To understand the vintage effect, a one-way analysis of variance (ANOVA), a principal component analysis (PCA), and a Partial Least Squares discriminant analysis (PLS-DA) were carried out. Data were divided into 66.6% to be used to train the model, and the remaining 33.3% employed to create the confusion matrix table. The one-way ANOVA results showed that there were significant differences in 21 PCs (*p* value ≤ 0.05) (Supplementary Table S4). No significant differences were found in delphinidin 3-*O*-glucoside (*p* value 0.08), delphinidin 3-*O*-acetylglucoside (*p* value 0.49), petunidin 3-*O*-acetylglucoside (*p* value 0.08), *p*-coumaric acid (*p* value 0.45), caffeic acid (*p* value 0.73) and quercetin (*p* value 0.13). The results of the PCA and PLS-DA (Fig. 2) show that the concentration and profiles of PCs were strongly dependent on the vintage with a clear separation over the 3 years of the study. Considering the compounds showing significant differences, the 2016 vintage was most associated with the levels of *trans-*piceid (also known as polydatin), ferulic acid, tyrosol, (−)-gallocatechin, (+)-catechin, syringic acid, *trans*-resveratrol and petunidin 3-*O*-glucoside. The 2018 vintage was associated to malvidin 3-*O*-glucoside, malvidin 3-*O*-acetylglucoside, malvidin 3-*O*-*p*-coumaroylglucoside, (−)-epicatechin, astilbin, peonidin 3-*O*-glucoside and cyanidin 3-*O*-glucoside. That is, different PCs were associated to the different vintages. After a cross-validation, the Balanced Error Rate of PLS-DA was 0 using ncomp = 4, thus achieving a good performance in the confusion matrix (Supplementary Table S5).

These results in the composition of wines show that the characteristics associated with terroir can only be resolved by including multiple vintages in the analysis. This is particularly necessary to avoid confusing terroir-specific effects with differences related to the growing season. For example, the 2017 and 2018 vintages showed climatic characteristics similar to the historical average of Mendoza^26^ and were reflected in increased levels of some PCs, particularly anthocyanins and flavonols. These responses were particularly evident for wines coming from GIs located at high elevations in the Uco Valley, where the levels for astilbin increased at least two-fold in comparison with 2016 (Supplementary Table S4). The 2016 vintage was exceptionally different since it was very rainy and humid, conditions that are not usual for Mendoza. In fact, the average rainfall during the summer of 2016 was 604 mm in comparison with a mean of 245 mm for the last 10 years (not published data obtained from weather stations of Catena Institute of Wine). The growing degree days (GDD) and rainfall data related to the climatic and geographical conditions of the studied sites is shown in Supplementary Table S6. The climate is probably the most important vintage-specific factor affecting berry quality at harvest. During the growing season is necessary to achieve a minimum cumulative temperature to ensure the complete ripening of certain varieties^15^. Heat accumulation that is too low during a given vintage results in delayed ripening, while heat accumulation that is too high promotes early ripening with lower berry quality^27^. The temperature amplitude between day and night favour accumulation of phenolic compounds, such as anthocyanins. The optimum berry temperature for anthocyanins synthesis during daytime at ripening stage is between 25 and 30 °C. In fact, temperatures above 35 °C stop anthocyanins accumulation or may even promote their degradation^27^. Our results are in agreement with those previously reported by Roullier-Gall et al.^1,6^. They showed different concentrations of PCs and other metabolites over 3 years for different terroirs of Burgundy and highlighting the importance of vintage on the composition of skins, musts, and wines. These results showed that while vintages have impact in grape characteristics, the most relevant dissimilarities are observed in grapes (skin and must) of different terroirs of a given vintage. Respect to wines, no significant terroir discrimination was achieved when wines were analysed immediately after elaboration. In contrast, when the same wines were analysed after bottle ageing a clear separation between closely related vineyards from the Côte de Beaune and the Côte de Nuits was achieved^6^.

The same authors suggested that the differences in concentrations of some specific compounds, such as *trans*-resveratrol, for a given vintage was a clear indication that the accumulation of PCs in berries must have been influenced by environmental factors, collectively referred as terroir conditions^1^. Anesi et al.^9^ evaluated the grapes composition of Corvina variety from 7 GIs over 3 years. They determined non-volatile and volatile compounds, highlighting that climate is probably the most important factor in the berries composition and quality. The authors suggested that the terroir effect can be measured only by having several years of study. In addition, they proposed statistical tools to avoid the vintage effect during the identification of distinct terroir signatures in the metabolic composition of berries from each macrozone. As well, Pereira et al.^7^ emphasised the prevalence of the vintage effect over soil characteristics on grape metabolic profiles. The research underline that the most important climatic characteristics are the seasonal sum of temperatures and water balance. These features define an individual vineyard and the characteristics of its grapes and wines.

### Correlation between phenolic composition and specific terroir features of GIs

**Figure 3:**
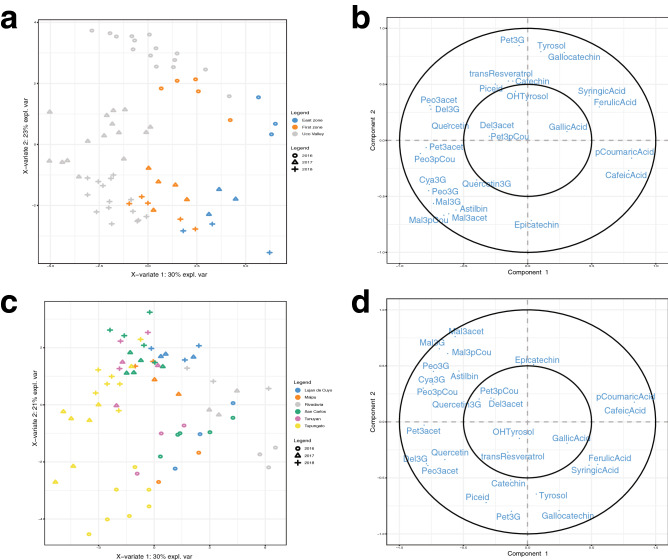
Supervised analysis with PLS-DA sample plot by (**a** and** b**) for Zones, and (**c** and** d**) for Departments. For comparative purposes all regions were classified as GIs. The figure was generated using Adobe Illustrator, version 22.1.0 (https://www.adobe.com/products/illustrator.html) and R-package mixOmics—‘mixOmics’.

**Figure 4:**
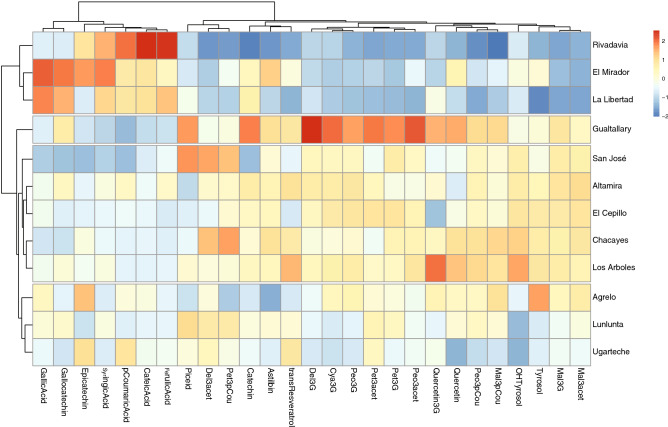
Hierarchical clustering of GIs (small areas) by phenolic composition. Values and cell colours indicate a relative concentration for each GI; blue corresponds to the minimum and red to the maximum value (“heatmap colours”). The figure was generated using Adobe Illustrator, version 22.1.0 (https://www.adobe.com/products/illustrator.html), R-package factoextra and R-package pheatmap—‘pheatmap’.

Data from the three vintages were used to carry out PLS-DA by using Zones (Fig. 3a,b) and Departments (Fig. 3 c and d) as classification variables. Zones are defined as the main viniculture areas (oasis) of Mendoza (East Zone, First Zone and Uco Valley; Supplementary Table 1). Departments are the political divisions of the province where the zones overlap. The GIs are smaller regions that include the parcels and were evaluated using a heatmap with cluster analysis (Fig. 4). As such, we evaluated the classification using different regional scales to know which is more or less predictable, and to understand the differences between the scales. As in the case of Zone and Departments, data were divided into 66.6% used to train the PLS-DA model, and 33.3%, employed to create the confusion matrix. The PLS-DAs were evaluated by the performance of a large number of components, using repeated fivefold cross-validation recurring 10 times for Zones and three-fold cross-validation recurring 10 times for Departments. The criteria of n-component used in the model was selected with the balanced classification error rate, because the number of samples per group was unbalanced, and the standard deviation agreed to prediction distances. The best performance was ncomp = 6 for Zones, ncomp = 7 for Departments.

Results of the two PLS-DAs showed a clear separation when Zones were used instead of Departments and GIs (Fig. 3). Although the classification variable was the location of parcels, the vintage was included to predict if wines come from the 2016, 2017 and 2018 vintages. As can be seen in Fig. 3, the 2016 harvest was clearly separated from 2017 and 2018, showing that the vintage effect was still present. The power of each variable in the first and second components is shown in Fig. S2, which indicates that the compounds ferulic acid, syringic acid, gallic acid, *p*-coumaric acid, caffeic acid, (−)-epicatechin and (+)-gallocatechin are associated with the East Zone, while the compounds petunidin 3-*O*-acetylglucoside, quercetin, cyanidin 3-*O*-glucoside, peonidin 3-*O*-acetylglucoside, peonidin 3-*O*-*p*-coumaroylglucoside, tyrosol, petunidin 3-*O*-glucoside, OH-tyrosol, astilbin, malvidin 3-*O*-*p*-coumaroylglucoside and quercetin-3-glucoside are related with the Uco Valley. It is important to mention that some compounds associated with the Uco Valley including tyrosol, petunidin 3-*O*-glucoside and OH-tyrosol seemed to be present in greater quantity in cool, wet years such as 2016. While astilbin, malvidin 3-*O*-*p* -coumarylglucoside and quercetin-3-glucoside had higher concentrations in the 2017 and 2018 vintages. Analysing the separation by region, the year effect seems to have a greater impact in areas such as the Uco Valley. In the case of the First Zone, only delphinidin 3-*O*-acetylglucoside was identified as a key variable that drives discrimination in components 1 and 2.

Confusion matrices were performed to test the PLS-DA models for each type of grouping and the Balanced Error Rate (BER) was calculated for each matrix. For the confusion matrix using Zones, the classification was good (BER 0.24). The area with lower true classes predicted was the East Zone; for a total of four observations, only two were predicted correctly, while two were predicted as the First Zone. For the Uco Valley, 94% of the observations used to test the model were correctly predicted, while one was predicted as the East Zone. In the case of the First Zone, 6 observations were used, five were correctly predicted and only one was incorrectly predicted as the Uco Valley.

In the confusion matrix using Departments (BER 0.49), Luján de Cuyo and Maipú could not be correctly predicted in any of the observations used to test the model. The observations of Tupungato were 88% correctly predicted. In the case of Rivadavia, the four observations were 100% correctly predicted. San Carlos had a good performance with four observations out of six correctly predicted.

A cluster analysis with heatmap using phenolic composition of the three vintages (2016, 2017 and 2018) was performed to identify the differences and similarities between the evaluated GIs. Figure 4 shows that Gualtallary (Tupungato Department; Uco Valley zone) is different respect to all others. Rivadavia, El Mirador, and La Libertad (Rivadavia Department; East Zone) form a different group. Ugarteche, and Agrelo (Luján de Cuyo Department; First Zone) form another group with Lunlunta (Maipú Departament; First Zone). The last group had two sub-groups and San José alone (Tupungato). Altamira and El Cepillo (San Carlos Department; Uco Valley Zone) presented very similar phenolic profiles and formed another subgroup. Los Árboles and Chacayes (Tunuyán Departament, Uco Valley Zone) formed a sub-group. As can be observed on the last group, the clustering by PCs is unclear for some GIs from San Carlos and Tunuyán departments, since very few exhibited correlations between their profiles and the distances between them (see map in Fig. 1). This analysis does not indicate that the wines from the two GIs from the same or nearby departments are identical, as could be initially supposed. In fact, the proximity in geographical distances between GIs is not a rule to indicate that wines from nearby areas may be similar, at least taking into account the PCs profile criteria. This may be also explained considering the variability in soil composition inside the same GI, which is strongly expressed in the final composition of wines.

The most interesting result was observed in Gualtallary. This GI It formed a cluster separated from the other GIs and it was characterized by high concentrations of delphinidin-3-*O*-glucoside, cyanidin-3-*O*-glucoside, peonidin-3-*O*-glucoside, petunidin-3-*O*-glucoside, peonidin-3-*O*-acetylglucoside and (+)-catechin. The contents of anthocyanins and other compounds, such as quercetin and *trans*-piceid, were higher in the more elevated GIs of the Uco Valley, particularly Gualtallary parcels. While considerably lower concentrations were observed in the Rivadavia Department (East Zone). The First Zone (Luján de Cuyo and Maipú Departments) showed an intermediate behaviour between the Uco Valley and the East Zone.

The concentration of PCs can be influenced by the elevation and temperatures of the places where vineyards are located. The elevation may influence the phenolic composition of the wine as a consequence of the higher levels of UV-B radiation and lower average temperatures^25,28,29^. Previous research has shown that UV-B radiation increases with elevation and was associated with increases in the concentration of quercetin, *trans*-resveratrol and other PCs such as di-hydroxylated anthocyanins^16,30^. Regarding anthocyanins, this research showed that the proportion of di-hydroxylated (cyanidin and peonidin) was considerably lower than tri-hydroxylated (delphinidin, petunidin, and malvidin) derivatives in GIs located at low elevations (Rivadavia; East Zone). On the other hand, GIs from the Uco Valley Zone exhibited the highest levels of anthocyanins. An increase in the relationship between di and tri-hydroxylated anthocyanins was directly associated with the elevation of GIs (See Fig. S1). The proportion of di and tri-hydroxylated anthocyanins was correlated with GDDs, mainly in 2017 and 2018 vintages (Fig. S1). The 2016 vintage had a very different behaviour in comparison to 2017 and 2018. The model showed strong differences in vintage variables (*p* value ≤ 0.001) and GDD (*p* value ≤ 0.01), having an interaction between both (*p* value ≤ 0.001). The results of *emmeans* package show that there are no significant differences between the slopes of the years 2017 and 2018 (*p* value 0.98), but both are different from the slope of the 2016 harvest (*p* value < 0.001).

As commented in “Introduction”, Mendoza is one of the few winemaking regions in the world where warm areas with Winkler Region V GDDs are only separated by less than 80 km from regions located in the Andes mountains characterized by colder climates with Winkler Region I and II GDDs (Fig. 1). The proximity to the mountains of GIs from the Uco Valley, was associated to their higher levels of PCs, particularly anthocyanins that are well associated with the separation in Fig. 4. Previous data supports that a cool climate tends to increase colour in red wines, whereas hotter day-time and night-time temperatures typically reduce, and even completely inhibit colouring^27^. As well, other research has shown that low nocturnal temperatures do not reverse the effects of higher day-time temperatures^27^. The optimal anthocyanin accumulation occurs when grapes are exposed to cool nights (15 °C) and moderate daytime temperatures (25 °C) during ripening. Schultz^31^ and Cozzolino et al.^32^ correlated low anthocyanin concentrations with warmer regions. The final anthocyanin concentration at high day-time temperatures seems to depend on the counterbalance between synthesis and degradation. De Rosas et al.^33^ observed similar results for Bonarda and Malbec berries of plants cultivated under field conditions. They observed a 40% average diminution of total anthocyanin content when berries grew under increased temperatures. These observations are in agreement with our results where GIs from the East zone were characterized by low levels of anthocyanins. As it is shown in Supplementary Table S6, the maximum and minimum temperatures as well as the number of days with more than 33 °C were considerably different. Azuma et al.^34^ showed that anthocyanin accumulation in grape berry skins is dependent on both low temperatures and light. They focused on flavonoid biosynthesis-related genes, observing a diminution of anthocyanin accumulation in 15 °C/low-light conditions, but the expression level of VlMYBA2, a light-responsive gene, was only slightly diminished. These results suggest that the final anthocyanin content in grape berry skin is determined not only by the expression levels of MYB related genes but also their modulation. Considering the different UV-B irradiances at high elevations for several of the studied GIs, particularly those of the Uco Valley, this is an additional factor related to the differential composition of PCs observed in Fig. 4. In China, Li et al.^16^ found a similar behaviour for Cabernet Sauvignon wines made with grapes grown at high elevations that had large differences in day and night temperatures, an annual sunshine time of 1987 h, and an annual rainfall of 300–600 mm. These climatic characteristics may stimulate the flow of more carbon towards the F3´H branch pathway. Consequently, quercetin derivatives and cyanidin-derived anthocyanins might accumulate at high quantities in berries, so explaining the high levels of these compounds in wines. Therefore, this ‘‘terroir” related condition might provide grapevines with a high activity of the F3´5´H branch pathway in the flavonoid metabolism of grape berries. As it is shown in Fig. 4, in addition to anthocyanins, quercetin and astilbin showed high levels in Gualtallary (the most elevated GI) in agreement with this previous report. In the case of the East Zone, the low concentration of anthocyanins compared to the wines obtained with grapes from the Uco Valley may be due to its chemical and/or enzymatic degradation. According to climatic data over the 3 years of study, the Rivadavia GI had up to 38 to 96 days with temperatures above 33 °C, while Gualtallary GI only had between 1 to 7 days. In a study with Cabernet Sauvignon, the loss of anthocyanins due to high temperatures, 35 °C between 6:00 a.m. and 8:00 p.m., halved their concentration as compared to 25 °C^36^. The exception was malvidin derivatives (3-glucoside, 3-acetylglucoside, and 3-*p*-coumaroylglucoside), since tri-substituted anthocyanins (particularly malvidin derivatives) were more abundant than their di-substituted counterparts in grape berries ripened under high-temperature conditions. As was explained previously and can be observed from Fig. 4, we obtained similar results for Malbec wines from different GIs in Mendoza. Particularly, cyanidin (di-substituted) derivatives were increased in high elevation (cooler) GIs. Another study evaluated the effects of two different temperature regimes on the accumulation of mRNAs and enzymes controlling berry skin anthocyanins on Sangiovese grapes^35^. The results showed that berries ripened under high temperatures (36 °C), a similar condition as the East and First zones of Mendoza, the biosynthesis of anthocyanins was suppressed at both, the transcriptional and enzymatic levels, but peroxidase activity was higher. They suggest that the low anthocyanin levels reflected the combined impact of reduced biosynthesis and increased degradation, particularly through the direct action of peroxidases in anthocyanin catabolism^35^.

### Potential for identifying unique parcels over different vintages

**Figure 5:**
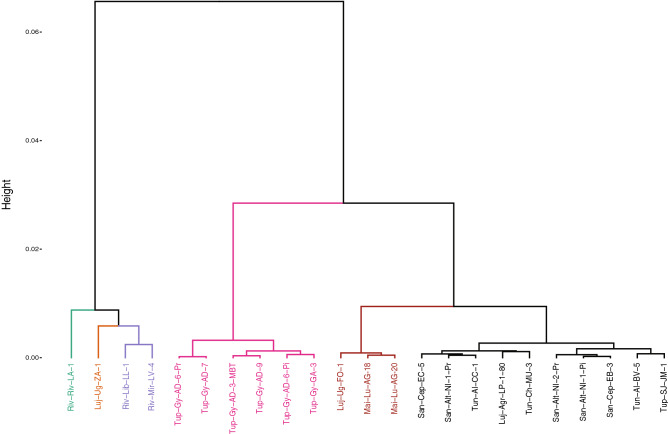
Cluster analysis with data from 23 Malbec parcels by using concentrations of PCs from three vintages (2016, 2017, and 2018). The figure was generated using Adobe Illustrator, version 22.1.0 (https://www.adobe.com/products/illustrator.html) and R-package factoextra and R-package factoextra—‘factoextra’.

To understand how parcels are classified based on their PCs profiles, a cluster analysis was performed using the integrated data from the three vintages. Figure 5 shows three large groups of parcels generated through this technique. The first group had four parcels, three of them belonged to the East Zone and one, the Luj-Ug-ZA-1, from Ugarteche GI, located in the First Zone. In the second group, all the parcels belong to Gualtallary GI. The parcels from this GI are located very close to each other and are similar in terms of climatic conditions, a relatively cold and high-elevated area (1350 to 1500 masl). As expected, each one of these parcels had similar chemical profiles because of climate similarities. Other authors also observed a similar behaviour in parcels of Shiraz in Australia^36^. In fact, the Gualtallary GI parcels had been selected for having very different soil characteristics^37^, yet with similar climatic conditions and at the same time for giving high quality and consistent wines over many years. The third group, showed a confounding grouping if we consider the parcel’s provenance. There was a sub-group formed by two wines from Lunlunta (Maipú Department) and one from Ugarteche (Luján de Cuyo Department). Another subgroup was formed by parcels that come from Agrelo (Luján de Cuyo Department), Altamira (San Carlos Department), El Cepillo (San Carlos Department), Chacayes (Tunuyán), Los Árboles (Tunuyán Department) and a parcel from San José (the lower elevation area of Tupungato department). According to these results, the groups that best relate wines with the origin include parcels from the East Zone (first group) and from the Gualtallary GIs (second group).

To achieve a predictive model for each parcel, a random forest analysis was chosen using the *randomForest* package in R. The random forest is a learning method for classification or regression that was proposed by Breiman^37^. These models have been used by various authors in the classification of wines and grapes from different regions^38,39^ to predicting aging of wines^41^, and even for identification of taxonomic features that explain the variation between sample conditions in microbial biodiversity patterns across viticulture zones^42^. Canizo et al.^40^ compared different data mining algorithms to study grape-skin samples of five regions in Mendoza. The results showed that data mining algorithms combined with multi-elemental analysis gave good geographical classification accuracy. Tian et al.^39^ also used random forest for the classification of wines from five wine regions of France using trace elements data, achieving a classification accuracy of 100% for all the tested wines. In our study, 66.6% of the data was used to train the model and the remaining 33.4% was used to test and create the confusion matrix. The results of the model showed an out of bag (OOB) estimate of error rate of 46.72% with ntree = 300. The results of the confusion matrix and statistics using the test data presented an accuracy of 0.746. The parcels with a correct classification (100%) were Luj-Ug-ZA-1, Mai-Lu-AG-20, Riv-Mir-LV-4, Riv-Riv-LA-1, San-Alt-NI-2-Pr, San-Cep-EB-3, Tun-Al-BV-5, Tun-Al-CC-1, Tun-Ch-MU-3, Tup-Gy-AD-6-Pi and Tup-Gy-AD-7. Random forest offers measures that can be used to get a ranking of relative importance of variables in the prediction. The most important variables were *p*-coumaric acid, delphinidin-3-*O*-glucoside, caffeic acid, quercetin and peonidin-3-*O*-glucoside. To our knowledge, the present work is the most extensive in terms of number of parcels evaluated across three different vintages for PCs in wines.

In this research, some of the parcels studied correspond to commercial wines. In this sense, knowing the fingerprint of each location is a potential tool for valorising high-quality wines, avoiding counterfeiting. Additionally, to know the characteristics of each place is useful for commercial communication or technical decisions during winemaking. In turn, consumers have learned to trust provenance to predict wine quality and are willing to pay an extra for it^38^. This type of wine marketing that relate wines with a specific place, has been carried out for many years in various wine regions of the world. This notion of terroir has demarcated many historical regions in Europe over the centuries, such as the famed vineyard sites of Burgundy, where their wines have included the concept of “climat”^43^. In Argentina, the expression "vino de parcela" has started to be used as a new classification to recognize wines made with grapes coming from small well characterized parcels. Therefore, the approach presented in this research helps to give some insights related to individualization of parcels with unique characteristics, a concept strongly related to terroir features of wines. Particularly interesting is the possibility of being able to identify such parcels over many years because of their consistency in phenolic profiles, especially those parcels associated with high quality wines.

## Conclusions

This study presented a comprehensive analysis of phenolic compounds of wines from 23 individual parcels, from Mendoza, Argentina. It aimed to discriminate wine characteristics based on terroir (locations) and across multiple vintages (2016, 2017 and 2018). A relationship between phenolic composition and climate conditions in the GIs studied was observed, highlighting the higher concentration of some specific PCs with low temperatures (GIs at high altitude). The results also suggest that, besides the vintage effect, some parcels can be correctly predicted independently of the year by using the phenolic profiles. In the light of these results, insights related to the individualization of parcels with unique characteristics have been proposed. Additionally, the possibility to identify those parcels associated with high quality wines and showing some consistency in phenolic profiles over multiple vintages will be of interest for the wine industry of Argentina. This fact will contribute to a better communication of the terroir characteristics of different regions and/or to make technical decisions during winemaking.

## Materials and methods

### Vineyard sites and winemaking procedures

Twenty-three parcels were selected in Mendoza province, distributed in 12 GIs belonging to six Departments in the three mains viticulture zones, First Zone, East Zone and Uco Valley (Fig. 1 and Table 1). The selection criteria for the parcels were: homogeneous soil within each parcel, own-rooted vines of more than 5 years old, and vineyard management using the same cultural practices. Detailed information of studied vineyards was summarized in Table 1. The study was carried out in the 2016, 2017 and 2018 vintages.

The winemaking of 201 microvinifications was carried out at the Catena Institute of Wine pilot-winery located in Agrelo, Luján de Cuyo, Mendoza in duplicate and triplicate for each parcel (See Table S7). Three out of twenty-three parcels were vinified only in duplicate, due to the small size of the parcels. Grapes were de-stemmed, then crushed, and the resulting must have transferred to 800 L plastic vessels for fermentation. At the time of incubation, 50 mg L^−1^ of SO_2_ (Enartis América Latina, Mendoza, Argentina) were added. After 24 h, 20 g L^−1^ Lavin EC-1118 (Lallemand Inc., Montréal, QC, Canada) active dry yeast were inoculated into the fermentation vessels. One day after inoculation, 100 mg L^−1^ of (NH_4_)_3_PO_4_ were added as the nitrogen source for the yeast. The fermentation temperature was 25 ± 2 °C, and °Brix and temperature monitored every 12 h. After alcoholic fermentation and 10 days of maceration, 50 L of drained wine were removed in the stainless-steel tanks. After 5 days of aging, 1 g L^−1^ of selected Lavin VP41 bacteria (Lallemand Inc., Montréal, Canada) was inoculated to perform the malolactic fermentation, which was considered complete when the malic acid content was below 0.2 g L^−1^ as assessed by OenoFoss (FOSS Analytical A/S, Hillerød, Denmark). After malolactic fermentation was finished, thick lees were removed by decantation. Afterwards, SO_2_ was added as K_2_S_2_O_5_ (Laffort Oenologie, France) to a final concentration of 35 mg L^−1^ free SO_2_. To maintain microbiological stability (avoiding deviations in malolactic fermentation and increase in volatile acidity) the pH was monitored keeping values below 3.79 by using tartaric acid (Derivados vínicos, Mendoza). Wines were stored for three months in 50 L stainless steel tanks at 13–15 °C. Finally, 48 green-glass bottles (750 mL volume) of each replicate (three per parcel) were fractionated and stored at 15 °C until analysis. Tin screw caps were used instead of natural cork as stoppers in order to prevent potential trichloroanisole contamination and/or variable oxygen incorporation.

### Basic oenological analysis of musts and wines

The initial parameters in must were measured on the same day of harvest and before crushing the grapes in the winery. Approximated 100 berries were hand-crushed and then the juice was used for the chemical parameter’s determination. The concentration of sugars (°Brix) was measured in the juice with a Pen-Harvest digital refractometer (Atago Co., Ltd., Tokyo, Japan). Total acidity, expressed as g L^−1^ of tartaric acid, was measured by titrating juice samples (10 mL) with 0.1 N NaOH to a final pH of 8.2. The standard chemical characteristics of must and wines were summarized in Table S7. Wine parameters including alcohol, total acidity, pH, volatile acidity and reducing sugars were analysed with the FTIR method using WineScan (FOSS, Hillerød, Denmark). The absorbance at 280, 420 and 520 nm were determined 1 month after bottling with a UV–VIS spectrophotometer Cary-50 (Varian Inc., Mulgrave, Australia) and quartz cuvettes of 1 mm pathlength. Wine colour intensity and hue were calculated by assessing the absorbance at 420 and 520 nm and calculating their ratio.

### TerraClimate data

Elevation (m a.s.l.), GDD and precipitation (rain, in mm) data for each parcel were obtained from the database of Catena Institute of Wine, Departmento de Agricultura y Contingencias Climáticas de Mendoza and WorldClim 2.1^44,45^ from October to April. The GDD were calculated using daily averages (in ºC) for the given periods and using a base of 10 °C^46^, while precipitation was calculated as the sum of the daily rain for the given periods.

### Chemicals

Standards of gallic acid (99%), 3-hydroxytyrosol (≥ 99.5%), (−)-gallocatechin (≥ 98%), (+)-catechin (≥ 99%), (−)-epicatechin (≥ 95%), dihydroquercetin 3-rhamnoside (astilbin) (≥ 98%), caffeic acid (99%), syringic acid (≥ 95%), *p-*coumaric acid (99%), ferulic acid (≥ 99%), *trans*-piceid) (≥ 95%), *trans*-resveratrol (≥ 99%), quercetin hydrate (95%), quercetin 3-β-d-glucoside (≥ 90%) and malvidin-3-*O*-glucoside chloride (≥ 95%) were purchased from Sigma-Aldrich. The standard of 2-(4-hydroxyphenyl) ethanol (tyrosol) (≥ 99.5%) was obtained from Fluka (Buchs, Switzerland). HPLC-grade acetonitrile (MeCN), methanol (MeOH) and formic acid (FA) were acquired from Mallinckrodt Baker Inc. (Pillispsburg, NJ, USA). Analytical grade sorbents (50 μm particle size) for dispersive solid phase extraction (d-SPE), including primary-secondary amine (PSA) and octadecylsilane (C_18_) were both obtained from Waters (Milford, MA, USA). Reagent grade NaCl, anhydrous MgSO_4_ and anhydrous CaCl_2_ were purchased from Sigma–Aldrich. Ultrapure water was obtained from a Milli-Q system (Millipore, Billerica, MA, USA). Individual stock solutions of compounds were prepared in MeOH at concentration levels ranging from 400 to 2000 µg mL^−1^. Further dilutions were prepared monthly in MeOH and stored in dark-glass bottles at -20 °C. Calibration standards used during optimization of HPLC–DAD conditions were dissolved in the initial mobile phase of the chromatographic method composed by ultrapure water (0.1% FA) and MeCN (95:5).

### Determination of PCs by HPLC–DAD

The determination of PCs was performed using a HPLC–DAD system (Dionex Softron GmbH, Thermo Fisher Scientific Inc., Germering, Germany). The instrument was a Dionex Ultimate 3000 with vacuum degasser unit, autosampler, quaternary pump, and chromatographic oven. A Dionex DAD-3000 (RS) detector with an analytical flow cell operated at a data collection rate of 5 Hz, a band width of 4 nm and a response time of 1.000 s was used. The wavelengths for quantification of the different families of LMW-PCs were 254 nm ((−)-gallocatechin and quercetin 3-β-d-glucoside), 280 nm (gallic acid, syringic acid, tyrosol, hydroxytyrosol, (+)-catechin, (−)-epicatechin and astilbin), 320 nm (caffeic acid, syringic acid, *p*-coumaric acid, ferulic acid, *trans*-piceid and *trans*-resveratrol) and 370 nm (quercetin). For anthocyanins, a wavelength of 520 nm was used. Acquisition parameters of the HPLC–DAD system and the data processing was performed by Chromeleon 7.1.

### Anthocyanins

The determination of anthocyanins was performed according to a previous methodology^47^, with minor modifications. A 500 μL aliquot of wine was evaporated to dryness and dissolved with 500 μL of initial mobile phase of anthocyanins HPLC method. The different anthocyanins were separated in a reversed-phase Kinetex C_18_ column (3.0 × 100 mm, 2.6 μm) Phenomenex (Torrance, CA, USA). The mobile phase consisted of ultrapure H_2_O:FA:MeCN (87:10:3, v/v/v; eluent A) and ultrapure H2O:FA:MeCN (40:10:50, v/v/v; eluent B) with the following gradient: 0 min, 10% B; 0–6 min, 25% B; 6–10 min, 31% B; 10–11 min, 40% B; 11–14 min, 50% B; 14–15 min, 100% B; 15–17 min, 10% B; 17–21 min, 10% B. The mobile phase flow was 1 mL min^−1^, column temperature 25 °C and injection volume 5 μL.

Quantification was carried out by measuring peak area at 520 nm and the content of each anthocyanin expressed as malvidin-3-glucoside equivalents using an external standard calibration curve (1–250 mg L^−1^, r^2^ = 0.997). The identity of detected anthocyanin compounds was confirmed by comparison with the elution profile and identification of analytes achieved in our previous research^48,49^.

### LMW-PCs

Compounds were extracted according to a sample preparation protocol previously published with minor modifications^47^. Briefly, 5 mL of wine were placed into a 15 mL PTFE centrifuge tube and acidified with FA (1%). Then, 2.5 mL MeCN were added and the tube hand-shaken 30 s. Phase separation was achieved by adding 1.5 g of NaCl and 4 g of MgSO_4_. The tubes were hand-shaken 1 min and centrifuged 10 min at 8000 rpm (6450 rcf). Afterwards, 1 mL aliquot of the upper MeCN phase was transferred to a 2 mL clean-up tube containing 150 mg CaCl_2_, 50 mg PSA and 50 mg C_18_ for dispersive solid-phase extraction (d-SPE). The mixture was vortexed 30 s and centrifuged 2 min at 12,000 rpm (8400 rcf). Lastly, an aliquot of 500 μL extract was evaporated to dryness under gentle N_2_ stream, the residue reconstituted with 500 μL of initial mobile phase and analysed by HPLC–DAD.

Chromatographic separations were carried out in reversed-phase Kinetex C_18_ column (3.0 × 100 mm, 2.6 µm) Phenomenex. The mobile phases were ultrapure water with 0.1% FA (A) and MeCN (B). Separation of analytes was performed using the following gradient: 0–1.7 min, 5% B; 1.7–10 min, 30% B; 10–13.5 min, 95% B; 13.5–15 min, 95% B; 15–16 min, 5% B: 16–19, 5% B. The mobile phase flow was 0.8 mL min^-1^. The column temperature was 35 °C and the injection volume 5 µL. LMW-PCs present in samples were quantified by using external calibration with pure authentic standards. Linear ranges between 0.25 and 40 mg L^−1^ with coefficient of determination (r^2^) higher than 0.998 were obtained for all the studied compounds.

### Data analysis

Data were analysed using software platform R 4.0.2 (R Foundation 3.1. for Statistical Computing, 2020)^48^. The PCs were analysed by Zones, Departments, parcels and vintages using one-way ANOVAs. Tukey honestly significant difference (HSD, 5% level) test was applied to PCs data to determine significant differences between the Zones, Departments, parcels and vintages. The relationship between di-hydroxylated and tri-hydroxylated compounds and GDD was analysed using a linear mixed-effect model with *lmer* function from the package *lme4*^49^and *lmerTest*^50^ with GDD and vintages (with interactions) as fixed factors, and nested parcels in the departments as random factors. To know if there were significant differences between the slopes of the vintages, the *emmeans* package was used in R^51^. PCA was applied to the wine phenolic composition to visualize the vintage effect using *FactoMineR* and *factoextra* packages^52,53^. The PLS-DA method was used to establish prediction models based on the anthocyanins and LMW-PCs content, the effect of vintage and regions (Zones andDepartments), using the *mixOmics* package^54^. Validation was performed using 66.6% of the data to train the model with the remaining data being employed to create the confusion matrix table. Balanced Error Rate (BER) was used to evaluate the classification performance, where the lower the error rate the better the performance. The heatmap used for parcel comparison was designed using *pheatmap* package^55^. Cluster analysis was constructed using Euclidean and Ward.D2 clustering technique and visualized using *FactoMineR* and *factomine* packages^52,53^. To find out which parcels can be predicted using PCs, Random Forest (RF) classification method was used with the *randomForest* package^56^. The validation and model test used 33% of the data not included in the initial model.

## Electronic supplementary material

Below is the link to the electronic supplementary material.

Supplementary Information 1.

## Data Availability

The datasets generated during and/or analysed during the current study are available from the corresponding author on reasonable request.
