# Supplementary material for: Terroir and vintage discrimination of Malbec wines based on phenolic composition across multiple sites in Mendoza, Argentina
Source: Sci Rep. 2021 Feb 3;11:2863. doi: 10.1038/s41598-021-82306-0 (PMC7859225; doi:10.1038/s41598-021-82306-0)
Supplement: Supplementary file 1 — Supplementary Information 1. [file 41598_2021_82306_MOESM1_ESM.pdf]

Terroir and vintage discrimination of Malbec wines based on phenolic composition across multiple sites in Mendoza, Argentina

Roy Urvieta, Gregory Jones, Fernando Buscema, Rubén Bottini, Ariel Fontana

Supplementary information

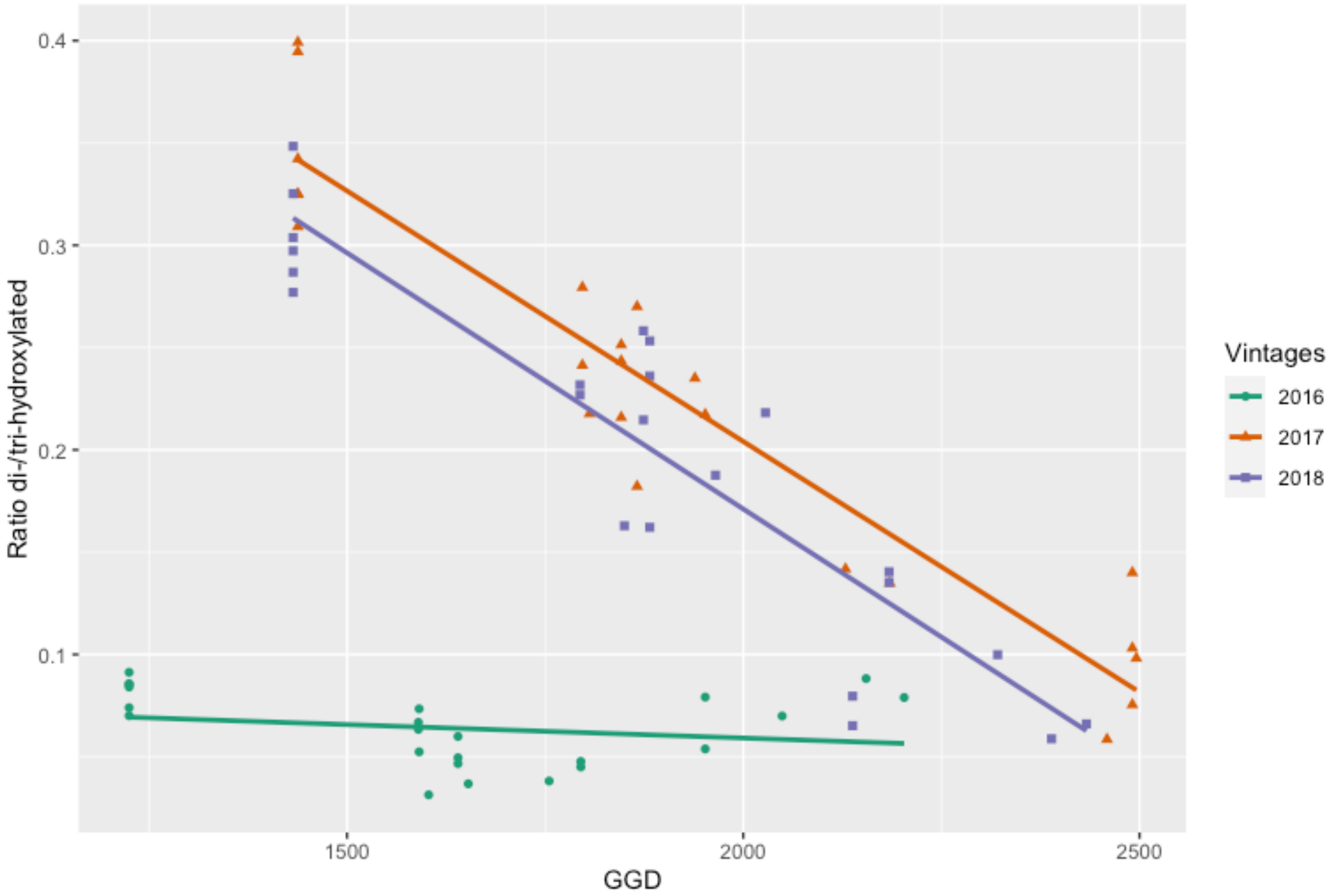

Figure S1. Relationship of GGD and ratio di-/tri-hydroxylated anthocyanins in three vintages. The images were created by PowerPoint and R-package emmeans - 'emmeans'

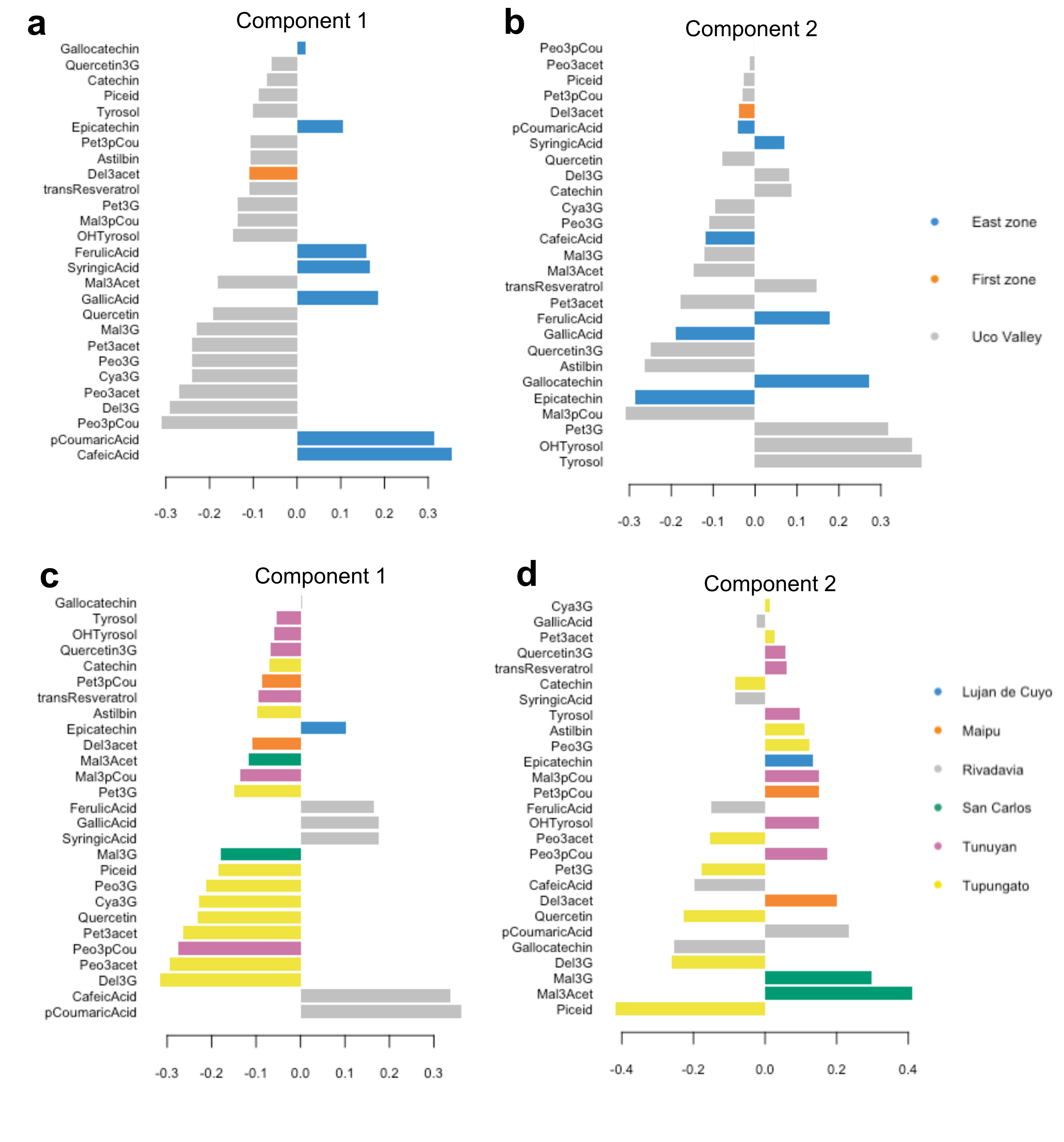

Figure S2. Loading plot of each feature selected on the first and second components of PLS-DA by Zones (a and b) and Departments (c and d), with color indicating the class with a maximal mean expression value for each PCs. The images were created by PowerPoint and R-package mixOmics - 'mixOmics'

Table S1. Overall data of anthocyanins and LMW-PCs (Name and PubChem CID) quantified [Mean (mg L−1) ± SD] in Malbec wines from different locations of Mendoza obtained in 2016 vintage.

		Anthocyanins											
Location		Delphinidin 3-O-glucoside - 102515359	Cyanidin 3-O-glucoside - 197081	Petunidin 3-O-glucoside - 443651	Peonidin 3-O-glucoside - 443654	Malvidin 3-O-glucoside - 443652	Delphinidin 3-O-acetylglucoside - 15385440	Petunidin 3-O-acetylglucoside - 44256961	Peonidin 3-O-acetylglucoside - 44256847	Malvidin 3-O-acetylglucoside - 72193646	Petunidin 3-O-*p*-coumaroylglucoside - 9897848	Peonidin 3-O-*p*-coumaroylglucoside - 131752297	Malvidin 3-O-*p*-coumaroylglucoside - 71308234
Parcels	Luj-Agr-LP-1-80	17.6 ± 4.7 ab	2.0 ± 0.2 ab	31.0 ± 22.9 cdefgh	6.6 ± 9.6 abc	306.6 ± 122.6 cdefgh	4.9 ± 0.8 abcd	8.5 ± 6.1 defghi	3.2 ± 2.5 bcdefg	32.6 ± 25.8 cdefg	3.7 ± 2.5 abcd	5.5 ± 2.9 defg	20.2 ± 2.9 efg
	Luj-Ug-FO-1	16.3 ± 4.3 abcd	1.5 ± 0.4 a	29.7 ± 5.7 cdefg	6.8 ± 7.4 abc	259.4 ± 23.4 bcde	11.1 ± 2.0 defg	10.20 ± 0.7 efghij	4.2 ± 0.8 efghi	24.8 ± 3.9 abcdef	6.6 ± 0.7 cdefgh	4.4 ± 0.6 bcdef	6.4 ± 3.0 abc
	Luj-Ug-ZA-1	4.7 ± 1.1 a	As	19.8 ± 1.6 abcd	13.7 ± 1.6 abcdef	307.0 ± 7.9 cdefgh	9.3 ± 1.6 abcdefg	3.5 ± 0.3 abcd	0.4 ± 0.2 abc	14.8 ± 3.1 abcd	0.6 ± 0.3 ab	0.9 ± 0.3 ab	14.5 ± 3.0 bcdef
	Mai-Lu-AG-18	16.7 ± 5.1 abcd	3.5 ± 1.1 bc	35.9 ± 4.3 defgh	13.3 ± 6.9 abcdef	360.6 ± 9.1 efghi	6.5 ± 2.6 abcde	7.1 ± 0.6 cdefgh	2.4 ± 0.2 abcdef	38.1 ± 4.3 efg	3.5 ± 0.5 abcd	6.9 ± 1.9 fghi	14.2 ± 1.1 bcdef
	Mai-Lu-AG-20	29.4 ± 1.5 de	5.4 ± 0.2 de	47.9 ± 2.3 ghij	22.8 ± 1.5 defg	360.2 ± 0.4 defghi	11.7 ± 1.2 efg	9.2 ± 0.1 efghij	3.4 ± 0.2 cdefg	41.2 ± 1.3 fg	5.3 ± 0.2 abcdef	9.8 ± 0.3 ijk	18.3 ± 1.8 def
	Riv-Lib-LL-1	12.1 ± 1.5 abc	1.7 ± 0.2 a	4.2 ± 1.6 ab	12.6 ± 9.9 abcde	163.4 ± 6.5 ab	4.3 ± 2.0 abc	1.2 ± 0.1 ab	0.3 ± 0.1 ab	7.4 ± 9.0 a	0.6 ± 0.2 ab	1.6 ± 0.6 abc	14.8 ± 2.7 bcdef
	Riv-Mir-LV-4	12.9 ± 1.8 abc	1.3 ± 0.1 a	7.3 ± 2.5 ab	13.6 ± 0.5 abcdef	212.9 ± 3.9 abc	4.1 ± 1.5 abc	2.1 ± 0.1 abc	0.5 ± 0 abcd	9.6 ± 1.3 ab	0.8 ± 0 ab	2.2 ± 0.2 abcd	1.6 ± 1.3 a
	Riv-Riv-LA-1	10.2 ± 0.8 abc	1.3 ± 0.1 a	0.9 ± 0.4 a	14.1 ± 1.3abcdef	145.7 ± 1.4 a	2.8 ± 0.7 a	0.8 ± 0 a	0.0 a	13.9 ± 1.1 abc	0.1 ± 0 a	0.7 ± 0.1 a	7.8 ± 0.5 abcd
	San-Alt-NI-1-Pi	21.4 ± 2.0 cd	3.4 ± 0.3 bc	43.5 ± 2.7 fghij	9.7 ± 1.6 abcd	382.5 ± 2.9 hi	6.4 ± 1.5 abcde	10.5 ± 0.3 fghij	3.5 ± 0.2 defgh	45.4 ± 0.5 fg	4.5 ± 0.3 abcdef	8.1 ± 0.4 ghij	14.0 ± 1.2 bcdef
	San-Alt-NI-1-Pr	17.9 ± 0.3 abcd	1.2 ± 0.2 a	11.8 ± 1.5 abc	12.8 ± 0.8 abcdef	254.6 ± 6.6 bcd	3.4 ± 0.6 ab	5.0 ± 0.2 abcde	1.5 ± 0.2 abcde	26.9 ± 3.0 abcdefg	1.8 ± 0.1 abc	4.0 ± 0.5 abcdef	4.3 ± 1.2 ab
	San-Alt-NI-2-Pr	7.6 ± 1.1 ab	1.0 ± 0.1 a	21.4 ± 4.0 abcde	10.5 ± 9.0 abcd	274.0 ± 38.6 cdefg	7.8 ± 1.9 abcdefg	5.3 ± 0.5 abcdef	1.7 ± 0.3 abcde	10.9 ± 4.1 ab	5.2 ± 0.6 abcdef	2.8 ± 0.6 abcde	7.1 ± 9.3 abc
	San-Cep-EB-3	42.7 ± 9.1 ef	6.2 ± 0.7 de	59.0 ± 7.1 ijk	18.8 ± 1.9 cdefg	357.8 ± 19.6 defghi	6.4 ± 3.0 abcde	12.8 ± 1.9 ij	5.4 ± 1.4 fghi	39.0 ± 4.3 fg	5.8 ± 0.9 bcdefg	9.2 ± 1.1 hijk	16.3 ± 2.9 cdef
	San-Cep-EC-5	19.4 ± 4.5 bcd	2.3 ± 0.3 ab	41.1 ± 5.9 efghi	15.9 ± 1.8 abcdef	366.6 ± 23.9 fghi	5.7 ± 2.0 abcde	10.0 ± 1.7 efghij	3.7 ± 1.0 efgh	46.0 ± 5.4 fg	4.1 ± 0.9 abcde	4.6 ± 0.9 cdefg	12.1 ± 4.1 abcdef
	Tun-Al-BV-5	27.4 ± 5.1 d	2.2 ± 0.6 ab	50.0 ± 2.5 ghij	13.4 ± 4.1 abcdef	372.6 ± 20.7 ghi	7.7 ± 1.6 abcdef	10.7 ± 1.9 ghij	6.6 ± 1.0 hijk	39.6 ± 3.7 fg	8.9 ± 3.7 defghij	12.2 ± 1.6 k	23.0 ± 3.9 fg
	Tun-Al-CC-1	7.4 ± 3.6 ab	0.8 ± 0.2 a	23.8 ± 5.3 bcdef	16.4 ± 1.1 bcdef	310.6 ± 28.0 cdefgh	6.2 ± 1.3 abcde	6.2 ± 1.3 bcdefg	3.6 ± 1.1 efgh	26.5 ± 4.1 abcdefg	9.4 ± 1.4 efghij	6.0 ± 0.8 efgh	11.1 ± 1.5 abcde
	Tun-Ch-MU-3	19.5 ± 2.9 bcd	1.2 ± 0.3 a	43.5 ± 3.4 fghij	1.4 ± 0.7 a	380.8 ± 0.9 hi	8.4 ± 2.4 abcdefg	11.5 ± 0.6 hij	6.3 ± 0.7 ghij	46.6 ± 3.5 g	7.5 ± 4.0 defghi	8.1 ± 0.2 ghij	22.8 ± 0.1 fg
	Tup-Gy-AD-3-MBT	83.8 ± 7.8 h	8.6 ± 1.2 f	97.3 ± 9.4 l	32.4 ± 3.7 g	452.7 ± 36.9 i	10.4 ± 2.8 cdefg	18.3 ± 0.5 k	10.4 ± 1.7 m	36.1 ± 3.5 defg	13.9 ± 2.9 j	12.6 ± 1.9 k	37.7 ± 4.7 h
	Tup-Gy-AD-6-Pi	47.2 ± 3.4 fg	5.7 ± 0.1 de	64.1 ± 0.2 jk	26.5 ± 0.2 efg	380.9 ± 16.0 hi	11.7 ± 2.8 efg	13.1 ± 0.8 ijk	7.0 ± 0.5 ijkl	29.8 ± 1.7 bcdefg	10.9 ± 0.6 ghij	10.3 ± 0.9 ijk	20.3 ± 1.5 efg
	Tup-Gy-AD-6-Pr	58.5 ± 6.1 g	6.7 ± 0.8 e	74.4 ± 4.0 k	27.3 ± 1.9 fg	373.0 ± 17.6 ghi	9.5 ± 0.5 bcdefg	14.3 ± 1.8 jk	9.5 ± 0.8 klm	27.7 ± 1.5 abcdefg	13.0 ± 1.0 ij	11.4 ± 0.8 jk	30.2 ± 0.8 gh
	Tup-Gy-AD-7	46.5 ± 3.3 fg	4.9 ± 0.6 cd	61.5 ± 6.0 ijk	14.7 ± 2.8 abcdef	318.7 ± 21.2 defgh	14.2 ± 2.6 fg	11.2 ± 0.2 ghij	8.6 ± 0.6 jklm	17.2 ± 2.3 abcde	9.9 ± 0.5 fghij	6.9 ± 0.6 fghi	18.8 ± 3.2 ef
	Tup-Gy-AD-9	49.8 ± 0.7 fg	5.1 ± 0.2 d	63.2 ± 2.0 jk	21.8 ± 1.2 defg	354.5 ± 11.8 defghi	14.4 ± 3.1 g	13.4 ± 0.2 ijk	9.9 ± 0.4 lm	26.2 ± 2.7 abcdefg	11.4 ± 0.3 hij	9.9 ± 0.8 ijk	22.1 ± 4.1 efg
	Tup-Gy-GA-3	46.6 ± 2.9 fg	6.0 ± 0.1 de	52.2 ± 2.6 hij	11.9 ± 0.8 abcde	262.2 ± 6.3 bcdef	6.2 ± 1.0 abcde	11.0 ± 0.1 ghij	6.6 ± 0.9 hijk	14.2 ± 0.6 abc	7.1 ± 3.1 cdefgh	5.9 ± 0.5 efgh	13.0 ± 1.4 bcdef
	Tup-SJ-JM-1	27.9 ± 6.0 d	1.7 ± 0.3 a	47.6 ± 6.8 ghij	3.3 ± 0.6 ab	354.1 ± 31.0 defghi	12.1 ± 3.8 efg	11.6 ± 1.8 hij	6.2 ± 1.4 ghij	33.3 ± 4.4 cdefg	5.5 ± 3.2 abcdefg	7.6 ± 0.9 fghi	17.1 ± 5.1 cdef
Departments	Lujan de Cuyo	12.9 ± 7.1 a	1.8 ± 0.3 a	26.8 ± 6.1 ab	9.0 ± 4.0 a	291.0 ± 27.3 ab	8.4 ± 3.2 ab	7.4 ± 3.5 ab	2.6 ± 2.0 ab	24.1 ± 8.9 ab	3.6 ± 3.0 ab	3.6 ± 2.4 ab	13.7 ± 6.9 a
	Maipu	23.0 ± 9.0 ab	4.4 ± 1.4 a	41.9 ± 8.5 bc	18.0 ± 6.7 a	360.4 ± 0.2 b	9.1 ± 3.6 ab	8.1 ± 1.5 ab	2.9 ± 0.7 ab	39.7 ± 2.2 b	4.4 ± 1.3 ab	8.4 ± 2.1 b	16.2 ± 2.9 a
	Rivadavia	11.7 ± 1.4 a	1.4 ± 0.3 a	4.2 ± 3.2 a	13.4 ± 0.8 a	174.0 ± 34.8 a	3.7 ± 0.8 a	1.4 ± 0.7 a	0.3 ± 0.2 a	10.3 ± 3.3 a	0.5 ± 0.4 a	1.5 ± 0.8 a	8.1 ± 6.6 a
	San Carlos	21.8 ± 12.8 ab	2.8 ± 2.1 a	35.4 ± 18.8 abc	13.5 ± 3.8 a	327.1 ± 58.4 b	6.0 ± 1.6 ab	8.7 ± 3.4 b	3.2 ± 1.6 ab	33.6 ± 14.9 ab	4.3 ± 1.5 ab	5.7 ± 2.8 ab	10.7 ± 4.9 a
	Tunuyan	18.1 ± 10.0 a	1.4 ± 0.7 a	39.1 ± 13.6 abc	10.4 ± 7.9 a	354.7 ± 38.4 b	7.4 ± 1.1 ab	9.5 ± 2.9 b	5.5 ± 1.6 bc	37.6 ± 10.2 b	8.6 ± 1.0 bc	8.8 ± 3.2 b	19.0 ± 6.8 a
	Tupungato	51.5 ± 16.9 b	5.5 ± 2.1 a	65.8 ± 16.4 c	19.7 ± 10.2 a	356.6 ± 58.4 b	11.2 ± 2.8 b	13.3 ± 2.5 b	8.3 ± 1.7 c	26.4 ± 8.0 b	10.2 ± 3.1 c	9.2 ± 2.5 b	22.7 ± 8.5 a
Zones	East zone	11.7 ± 1.4 a	1.4 ± 0.3 a	4.2 ± 3.2 a	13.4 ± 0.8 a	174.0 ± 34.8 a	3.7 ± 0.8 a	1.4 ± 0.7 a	0.3 ± 0.2 a	10.3 ± 3.3 a	0.5 ± 0.4 a	1.5 ± 0.8 a	8.1 ± 6.6 a
	First zone	16.9 ± 8.8 a	2.8 ± 1.6 a	32.9 ± 10.3 ab	12.6 ± 6.6 a	318.8 ± 42.6 b	8.7 ± 2.9 b	7.7 ± 2.6 b	2.7 ± 1.4 ab	30.3 ± 10.7 b	3.9 ± 2.2 ab	5.5 ± 3.3 ab	14.7 ± 5.3 a
	Uco Valley	34.9 ± 21.0 a	3.8 ± 2.5 a	50.3 ± 21.6 b	15.8 ± 8.6 a	346.4 ± 53.3 b	8.7 ± 3.3 b	11.0 ± 3.5 b	6.0 ± 2.8 b	31.0 ± 11.3 b	7.9 ± 3.5 b	8.0 ± 3.0 b	18.0 ± 8.6 a

		LMW-PCs													
Location		Gallic acid - 370	OH-tyrosol - 82755	Tyrosol - 10393	(+)-Catechin - 9064	Syringic acid - 10742	(-)-Epicatechin - 72276	Astilbin - 119258	Caffeic acid - 689043	*p*-coumaric acid - 637542	Ferulic acid - 445858	*trans*-Piceid - 5281718	*trans*-resveratrol - 445154	Quercetin-3-glucoside - 5280804	Quercetin - 5280343	(-)-Gallocatechin - 9882981
Parcels	Luj-Agr-LP-1-80	27.0 ± 4.2 def	3.8 ± 0.8 ghi	37.7 ± 11.6 cdef	21.5 ± 6.9 abc	16.3 ± 3.5 d	23.3 ± 4.3 bcdef	4.6 ± 1.1 abcd	11.9 ± 2.6 fg	23.3 ± 6.2 de	1.3 ± 0.5 abc	0.7 ± 0.3 a	4.4 ± 1.0 abc	0.8 ± 0.1 ab	8.8 ± 0.6 bcdefg	11.4 ± 1.8 ab
	Luj-Ug-FO-1	27.6 ± 4.3 efg	2.2 ± 0.1 abcde	37.8 ± 1.9 cdef	29.3 ± 5.4 c	9.1 ± 1.2 ab	23.8 ± 2.5 bcdef	6.9 ± 1.1 cdefg	5.1 ± 1.4 abcde	18.6 ± 4.1 bcd	1.0 ± 0.8 ab	5.5 ± 2.0 cdef	6.2 ± 1.0 bcde	0.5 ± 0 a	8.8 ± 2.0 bcdefg	10.8 ± 1.7 ab
	Luj-Ug-ZA-1	15.6 ± 0.5 ab	2.6 ± 0.4 abcdefg	15.5 ± 0.2 ab	13.1 ± 0.8 ab	13.2 ± 0.3 bcd	19.6 ± 1.0 abc	3.4 ± 0.4 abc	12.8 ± 2.3 g	39.6 ± 6.0 f	1.1 ± 0.2 ab	0.1 ± 0 a	4.5 ± 0.7 abc	0.7 ± 0.3 ab	2.5 ± 0.6 a	10.4 ± 1.1 ab
	Mai-Lu-AG-18	30.2 ± 4.4 fgh	2.0 ± 0.3 abcd	28.1 ± 2.5 bcd	27.9 ± 10.8 bc	14.9 ± 1.2 cd	29.7 ± 7.9 def	2.8 ± 1.1 ab	13.6 ± 1.3 g	21.1 ± 4.1 cd	1.0 ± 0.2 ab	2.5 ± 0.5 abcd	5.7 ± 0.3 abcde	0.7 ± 0.1 ab	6.1 ± 1.0 abcd	17.3 ± 3.0 bcde
	Mai-Lu-AG-20	26.2 ± 3.5 def	1.3 ± 0.2 ab	30.1 ± 5.7 cde	24.5 ± 5.3 abc	12.0 ± 1.0 abcd	20.3 ± 2.9 abcd	6.0 ± 2.3 bcdef	6.5 ± 0.5 cde	14.4 ± 1.1 abcd	1.0 ± 0 ab	5.4 ± 1.5 cdef	3.2 ± 0.1 ab	0.9 ± 0.1 ab	13.9 ± 0.6 gh	11.8 ± 3.0 abc
	Riv-Lib-LL-1	51.5 ± 3.6 i	3.7 ± 0.9 fghi	10.2 ± 2.5 a	20.5 ± 4.5 abc	27.0 ± 1.9 e	23.4 ± 3.1 bcdef	4.0 ± 0.8 abc	24.7 ± 0.8 i	39.4 ± 1.3 f	2.8 ± 0.3 d	2.2 ± 1.0 abcd	3.1 ± 0.1 ab	0.6 ± 0 a	6.7 ± 1.5 abcde	19.4 ± 4.8 de
	Riv-Mir-LV-4	35.2 ± 3.3 gh	4.5 ± 0.8 hi	27.2 ± 1.6 bcd	29.6 ± 2.5 c	26.8 ± 2.8 e	26.4 ± 1.1 cdef	4.8 ± 0.6 abcde	34.8 ± 3.1 k	36.0 ± 11.2 ef	2.1 ± 0.4 bcd	0.7 ± 1.0 a	5.3 ± 1.3 abcde	0.6 ± 0 a	7.4 ± 1.6 abcdef	18.5 ± 4.2 cde
	Riv-Riv-LA-1	26.9 ± 3.2 def	4.0 ± 0.1 ghi	7.1 ± 1.1 a	10.6 ± 1.7 a	29.6 ± 1.0 e	19.5 ± 1.9 abc	2.1 ± 0.2 a	30.3 ± 1.7 j	46.5 ± 6.9 f	2.6 ± 0.2 cd	0.4 ± 0.2 a	4.0 ± 0.3 abc	0.6 ± 0 a	6.5 ± 1.0 abcde	17.3 ± 0.6 bcde
	San-Alt-NI-1-Pi	16.6 ± 2.0 abc	2.7 ± 0 bcdefg	39.3 ± 3.6 cdef	20.5 ± 2.3 abc	14.8 ± 0.2 cd	20.3 ± 1.9 abcd	6.4 ± 0.7 bcdef	6.7 ± 0.1 cde	20.9 ± 0.6 cd	1.1 ± 0.1 ab	0.9 ± 0.1 ab	5.6 ± 0.7 abcde	1.3 ± 0 b	7.2 ± 0.5 abcdef	14.1 ± 0.9 abcd
	San-Alt-NI-1-Pr	23.9 ± 1.0 cdef	3.6 ± 0.5 efghi	36.8 ± 2.8 cdef	31.7 ± 3.5 c	13.4 ± 0.9 bcd	30.2 ± 3.4 ef	3.8 ± 0.2 abc	8.3 ± 1.0 ef	17.8 ± 0.5 bcd	1.0 ± 0.1 ab	0.2 ± 0 a	5.2 ± 0.3 abcd	0.6 ± 0 a	4.2 ± 0.4 ab	15.8 ± 3.3 abcde
	San-Alt-NI-2-Pr	22.2 ± 0.3 bcdef	3.3 ± 0.1 defghi	25.4 ± 2.0 bc	28.1 ± 1.3 c	13.6 ± 2.0 bcd	28.0 ± 0.6 cdef	4.2 ± 1.0 abc	18.7 ± 1.0 h	43.4 ± 2.7 f	1.8 ± 0 abcd	0.3 ± 0.1 a	6.8 ± 1.0 cde	0.6 ± 0.1 a	5.2 ± 0.8 abc	12.7 ± 0.9 abcd
	San-Cep-EB-3	21.7 ± 2.8 bcde	2.9 ± 0.5 cdefg	37.8 ± 4.4 cdef	24.3 ± 6.4 abc	13.5 ± 0.8 bcd	21.2 ± 5.7 abcde	3.7 ± 2.0 abc	8.1 ± 1.0 def	17.8 ± 1.8 bcd	0.9 ± 0.2 ab	0.6 ± 0.4 a	6.8 ± 1.8 cde	1.0 ± 0.5 ab	11.5 ± 6.7 defgh	13.2 ± 3.3 abcd
	San-Cep-EC-5	19.3 ± 1.4 abcd	2.6 ± 0.4 bcdefg	45.1 ± 10.0 f	26.6 ± 3.2 bc	12.5 ± 1.8 abcd	19.2 ± 1.6 abc	6.7 ± 0.7 cdefg	5.0 ± 1.0 abcde	13.9 ± 1.4 abcd	0.6 ± 0.1 a	1.0 ± 0.2 ab	2.7 ± 0.5 a	0.6 ± 0.1 ab	7.7 ± 0.9 abcdef	10.3 ± 0.8 ab
	Tun-Al-BV-5	15.9 ± 1.8 abc	2.3 ± 0.7 abcdef	41.2 ± 2.1 def	28.7 ± 2.1 c	11.6 ± 1.1 abc	14.9 ± 2.5 ab	8.9 ± 1.6 fg	1.9 ± 1.5 ab	7.4 ± 4.4 ab	1.1 ± 0.1 ab	3.7 ± 3.5 abcde	8.6 ± 2.9 efg	0.9 ± 0.3 ab	13.6 ± 0.5 gh	12.7 ± 1.1 abcd
	Tun-Al-CC-1	15.7 ± 0.6 ab	4.5 ± 0.6 i	40 ± 2.1 def	21.6 ± 4.0 abc	13.3 ± 1.1 bcd	15.6 ± 0.8 ab	7.1 ± 0.2 cdefg	6.1 ± 1.5 bcde	20.9 ± 0.1 cd	1.0 ± 0.2 ab	0.4 ± 0.1 a	6.9 ± 0.9 cdef	0.9 ± 0.1 ab	12.8 ± 1.3 fgh	12.0 ± 0.4 abc
	Tun-Ch-MU-3	15.0 ± 2.8 ab	2.3 ± 0 abcdef	39.1 ± 2.3 cdef	25.7 ± 11.3 bc	11.3 ± 0.7 abc	15.5 ± 2.9 ab	7.1 ± 3.3 cdef	4.5 ± 0.7 abcde	17.7 ± 2.8 bcd	0.9 ± 0.1 ab	1.1 ± 0.3 ab	5.9 ± 0.2 abcde	0.6 ± 0.1 a	10.7 ± 0.6 cdefgh	9.0 ± 1.5 a
	Tup-Gy-AD-3-MBT	19.4 ± 1.3 abcd	1.6 ± 0.3 abc	32.5 ± 2.1 cdef	22.8 ± 0.6 abc	9.1 ± 0.8 ab	14.6 ± 1.7 ab	9.2 ± 1.1 fg	2.6 ± 0.8 abc	6.3 ± 0.6 ab	0.6 ± 0.1 a	7.4 ± 0.7 f	6.9 ± 1.4 cdef	0.5 ± 0 a	12.4 ± 0.9 efgh	14.8 ± 0.4 abcd
	Tup-Gy-AD-6-Pi	19.3 ± 1.9 abcd	1.6 ± 0.3 abc	39.1 ± 3.3 cdef	31.4 ± 3.4 c	9.6 ± 0.4 ab	16.4 ± 2.2 ab	8.5 ± 0.7 efg	3.9 ± 0.2 abcd	10.0 ± 0.3 abc	0.8 ± 0 ab	2.2 ± 0.2 abcd	10.6 ± 1.7 g	0.6 ± 0 a	9.6 ± 1.2 bcdefgh	14.6 ± 0.6 abcd
	Tup-Gy-AD-6-Pr	16.2 ± 0.5 abc	1.1 ± .1 a	39.3 ± 5.3 cdef	22.4 ± 1.1 abc	9.0 ± 0.5 ab	13.5 ± 0.4 a	9.5 ± 0.2 fg	0.9 ± 0.5 a	4.2 ± 1.7 a	0.7 ± 0.1 a	7.3 ± 1.7 ef	8.2 ± 0.5 defg	0.6 ± 0.1 a	10.1 ± 0.9 bcdefgh	14.5 ± 0.5 abcd
	Tup-Gy-AD-7	12.8 ± 0.1 a	1.5 ± 0 abc	31.8 ± 4.3 cdef	17.8 ± 2.3 abc	10.0 ± 1.2 ab	12.7 ± 0.5 a	9.3 ± 1.1 fg	1.4 ± 0.3 a	4.2 ± 0.3 a	1.4 ± 0.4 abc	5.3 ± 1.6 cdef	2.8 ± 0.5 ab	0.9 ± 0.1 ab	15.3 ± 2.9 h	13.6 ± 2.0 abcd
	Tup-Gy-AD-9	15.1 ± 0.4 ab	1.3 ± 0.1 ab	39.3 ± 2.8 cdef	19.8 ± 1.3 abc	8.3 ± 0.2 a	14.7 ± 0.7 ab	6.8 ± 0.7 cdefg	1.9 ± 0.4 ab	6.2 ± 2.0 ab	0.6 ± 0.1 a	4.4 ± 0.6 bcdef	10.3 ± 0.2 fg	0.6 ± 0 ab	12.2 ± 0.3 efgh	12.8 ± 0.5 abcd
	Tup-Gy-GA-3	37.3 ± 2.4 h	3.1 ± 0.2 defgh	42.4 ± 1.1 ef	58.3 ± 2.3 d	8.4 ± 0.5 a	32.0 ± 2.4 f	8.2 ± 0.6 defg	6.0 ± 0.4 bcde	3.1 ± 0.5 a	0.5 ± 0 a	2.1 ± 0.3 abc	7.0 ± 0.4 cdef	0.6 ± 0.1 a	14.0 ± 0.9 gh	22.7 ± 0.7 e
	Tup-SJ-JM-1	17.9 ± 1.1 abc	2.0 ± 0 abcd	38.5 ± 2.1 cdef	24.5 ± 5.7 abc	10.6 ± 1.1 abc	15.1 ± 1.6 ab	10.3 ± 1.5 g	4.6 ± 0.5 abcde	6.8 ± 1.2 ab	1.6 ± 1.5 abcd	5.8 ± 1.0 def	6.6 ± 0.5 cde	0.9 ± 0.6 ab	10.1 ± 1.4 cdefgh	11.1 ± 2.2 ab
Departments	Lujan de Cuyo	23.4 ± 6.7 ab	2.9 ± 0.9 ab	30.3 ± 12.8 ab	21.3 ± 8.1 a	12.8 ± 3.6 ab	22.2 ± 2.3 a	5.0 ± 1.7 ab	9.9 ± 4.2 a	27.1 ± 11.0 bc	1.1 ± 0.1 a	2.1 ± 3.0 a	5.0 ± 1.0 a	0.7 ± 0.1 a	6.7 ± 3.7 a	10.9 ± 0.5 a
	Maipu	28.2 ± 2.8 ab	1.6 ± 0.5 a	29.1 ± 1.4 ab	26.2 ± 2.4 a	13.4 ± 2.0 b	25.0 ± 6.6 a	4.4 ± 2.2 ab	10.0 ± 5.1 a	17.7 ± 4.7 ab	1.0 ± 0.1 a	3.9 ± 2.0 a	4.5 ± 1.8 a	0.8 ± 0.1 a	10.0 ± 5.5 a	14.6 ± 3.9 ab
	Rivadavia	37.9 ± 12.5 b	4.1 ± 0.4 b	14.8 ± 10.8 a	20.2 ± 9.5 a	27.8 ± 1.6 c	23.1 ± 3.5 a	3.6 ± 1.4 a	29.9 ± 5.0 b	40.6 ± 5.4 c	2.5 ± 0.4 b	1.1 ± 1.0 a	4.1 ± 1.1 a	0.6 ± 0 a	6.9 ± 0.5 a	18.4 ± 1.1 b
	San Carlos	20.7 ± 2.8 ab	3.0 ± 0.4 ab	36.9 ± 7.2 b	26.2 ± 4.2 a	13.6 ± 0.8 b	23.8 ± 5.0 a	5.0 ± 1.5 ab	9.3 ± 5.4 a	22.8 ± 11.8 abc	1.1 ± 0.5 a	0.6 ± 0.3 a	5.4 ± 1.7 a	0.8 ± 0.3 a	7.2 ± 2.8 a	13.2 ± 2.0 ab
	Tunuyan	15.5 ± 0.5 a	3.1 ± 1.3 ab	40.1 ± 1.0 b	25.3 ± 3.6 a	12.1 ± 1.1 ab	15.3 ± 0.4 a	7.7 ± 1.0 bc	4.2 ± 2.1 a	15.3 ± 7.0 ab	1.0 ± 0.1 a	1.7 ± 1.8 a	7.1 ± 1.4 a	0.8 ± 0.2 a	12.4 ± 1.5 a	11.2 ± 2.0 a
	Tupungato	19.7 ± 8.1 a	1.7 ± 0.6 a	37.6 ± 3.9 b	28.1 ± 14.0 a	9.3 ± 0.8 a	17.0 ± 6.7 a	8.8 ± 1.1 c	3.0 ± 1.8 a	5.8 ± 2.3 a	0.9 ± 0.4 a	5.0 ± 2.2 a	7.5 ± 2.6 a	0.7 ± 0.1 a	12.0 ± 2.2 a	14.9 ± 3.7 ab
Zones	East zone	37.9 ± 12.5 b	4.1 ± 0.4 b	14.8 ± 10.8 a	20.2 ± 9.5 a	27.8 ± 1.6 b	23.1 ± 3.5 a	3.6 ± 1.4 a	29.9 ± 5.0 b	40.6 ± 5.4 b	2.5 ± 0.4 b	1.1 ± 1.0 a	4.1 ± 1.1 a	0.6 ± 0 a	6.9 ± 0.5 a	18.4 ± 1.1 b
	First zone	25.3 ± 5.6 a	2.4 ± 0.9 a	29.8 ± 9.1 b	23.3 ± 6.4 a	13.1 ± 2.8 a	23.3 ± 4.0 a	4.7 ± 1.7 ab	10.0 ± 3.9 a	23.4 ± 9.6 a	1.1 ± 0.1 a	2.8 ± 2.5 a	4.8 ± 1.2 a	0.7 ± 0.1 a	8.0 ± 4.2 a	12.4 ± 2.8 a
	Uco Valley	19.2 ± 5.9 a	2.4 ± 1.0 a	37.8 ± 4.8 b	26.9 ± 9.6 a	11.3 ± 2.2 a	18.9 ± 6.3 a	7.3 ± 2.1 b	5.4 ± 4.4 a	13.4 ± 10.5 a	1.0 ± 0.4 a	2.9 ± 2.6 a	6.7 ± 2.2 a	0.7 ± 0.2 a	10.4 ± 3.2 a	13.6 ± 3.1 a

Different letters within the same row indicate significant differences ($p < 0.05$) according to a Tukey HSD test.

Table S2. Overall data of anthocyanins and LMW-PCs (Name and PubChem CID) quantified [Mean (mg L−1) ± SD] in Malbec wines from different locations of Mendoza obtained in 2017 vintage.

Location		Anthocyanins											
		Delphinidin 3-O-glucoside - 102515359	Cyanidin 3-O-glucoside - 197081	Petunidin 3-O-glucoside - 443651	Peonidin 3-O-glucoside - 443654	Malvidin 3-O-glucoside - 443652	Delphinidin 3-O-acetylglucoside - 15385440	Petunidin 3-O-acetylglucoside - 44256961	Peonidin 3-O-acetylglucoside - 44256847	Malvidin 3-O-acetylglucoside - 72193646	Petunidin 3-O-*p*-coumaroylglucoside - 9897848	Peonidin 3-O-*p*-coumaroylglucoside - 131752297	Malvidin 3-O-*p*-coumaroylglucoside - 71308234
	Luj-Agr-LP-1-80	17.2 ± 0.5 abc	81.9 ± 6.7 efg	6.2 ± 0.1 abcde	135.8 ± 10.3 efg	779.6 ± 48.8 cde	9.8 ± 11.6 ab	8.8 ± 1.2 abcde	2.9 ± 0.0 ab	107.9 ± 4.2 def	8.3 ± 11.7 abcdef	10.8 ± 0.9 abcd	46.6 ± 6.7 cdef
	Luj-Ug-FO-1	9.4 ± 1.4 ab	37.4 ± 2.4 abcde	3.9 ± 0.2 abc	79.3 ± 3.2 cd	708.0 ± 9.6 bc	13.8 ± 2.5 ab	17.9 ± 0.6 fg	3.4 ± 0.1 abcd	97.9 ± 2.3 cde	12.3 ± 0.2 bcdef	8.7 ± 0.2 abcd	45.4 ± 1.5 cdef
	Luj-Ug-ZA-1	8.6 ± 1.4 ab	37.0 ± 1.6 abcd	2.6 ± 0.6 ab	78.7 ± 1.2 cd	764.9 ± 15.8 cd	5.7 ± 0.2 ab	12.2 ± 0.4 abcdefg	4.1 ± 0.2 abcd	97.9 ± 7.7 cde	10.6 ± 0.8 abcdef	6.4 ± 0.4 abc	33.1 ± 1.0 abcd
	Mai-Lu-AG-18	13.5 ± 5.6 abc	32.6 ± 11.3 abc	4.3 ± 1.1 abc	69.0 ± 11.8 bcd	625.2 ± 42.4 b	14.5 ± 3.6 ab	14.6 ± 2.5 cdefg	3.0 ± 0.6 ab	86.4 ± 7.9 bc	12.6 ± 1.2 bcdef	9.8 ± 1.1 abcd	43.0 ± 4.3 cde
	Mai-Lu-AG-20	5.2 ± 1.5 a	13.0 ± 2.6 ab	3.0 ± 0.3 abc	41.8 ± 4.4 abc	476.3 ± 17.9 a	10.0 ± 0.8 ab	9.0 ± 0.6 abcde	2.7 ± 0.3 ab	70.0 ± 2.4 b	11.5 ± 0.6 bcdef	7.5 ± 0.5 abcd	44.1 ± 1.8 cde
	Riv-Lib-LL-1	9.9 ± 7.8 ab	5.4 ± 5.4 a	2.5 ± 0.7 ab	24.3 ± 6.7 a	360.1 ± 35.2 a	6.5 ± 0.2 ab	5.5 ± 1.2 ab	1.0 ± 0.4 a	36.5 ± 3.4 a	5.5 ± 1.0 abcd	3.3 ± 0.7 a	19.0 ± 3.9 a
	Riv-Mir-LV-4	6.2 ± 9.8 a	8.5 ± 1.6 ab	2.6 ± 0.2 ab	32.6 ± 2.2 ab	392.0 ± 17.5 a	3.7 ± 0.9 ab	6.2 ± 0.9 abc	2.8 ± 2.1 ab	48.5 ± 6.1 a	9.0 ± 0.5 abcdef	5.4 ± 0.4 ab	33.0 ± 3.0 abc
	Riv-Riv-LA-1	6.7 ± 1.0 a	11.8 ± 2.9 ab	1.2 ± 0.1 a	10.7 ± 4.3 a	364.3 ± 22.2 a	2.5 ± 0.7 ab	4.8 ± 0.6 a	1.1 ± 0.1 a	45.7 ± 4.6 a	4.9 ± 0.8 abc	3.3 ± 0.4 a	24.8 ± 2.7 ab
	San-Alt-NI-1-Pi	41.2 ± 2.7 efg	73.4 ± 1.7 cdefg	8.8 ± 0.4 abcdef	122.1 ± 1.9 ef	768.8 ± 6.4 cd	12.7 ± 1.7 ab	4.8 ± 0.6 a	3.4 ± 0.3 abc	92.1 ± 2.0 cde	11.7 ± 0.5 bcdef	11.7 ± 0.8 abcd	35.5 ± 1.0 bcd
	San-Alt-NI-1-Pr	50.8 ± 2.2 ghi	94.3 ± 6.9 fg	9.4 ± 0.4 abcdef	148.7 ± 7.6 fgh	803.9 ± 32.5 cde	12.6 ± 7.0 ab	9.2 ± 1.2 abcdef	4.5 ± 0.6 abcd	97.7 ± 4.7 cde	14.9 ± 1.1 def	14.0 ± 0.6 bcd	41.7 ± 3.7 cde
	San-Alt-NI-2-Pr	63.1 ± 5.1 ij	99.6 ± 17.0 g	10.2 ± 1.1 bcdef	156.0 ± 18.0 fghi	866.0 ± 44.9 de	11.1 ± 7.8 ab	10.3 ± 2.5 abcdef	4.5 ± 0.7 abcd	110.6 ± 4.5 ef	15.4 ± 1.9 ef	16.5 ± 1.8 d	45.6 ± 2.2 cdef
Parcels	San-Cep-EB-3	35.9 ± 7.1 defg	117.4 ± 16.0 ghi	11.2 ± 1.8 cdef	167.9 ± 13.2 ghi	842.1 ± 30.8 de	13.1 ± 3.3 ab	14.0 ± 2.0 bcdefg	5.9 ± 1.2 bcd	109.7 ± 5.8 def	15.2 ± 1.1 def	10.0 ± 1.0 abcd	35.2 ± 3.0 bcd
	San-Cep-EC-5	25.6 ± 3.9 bcde	88.0 ± 11.7 fg	7.5 ± 0.6 abcdef	143.8 ± 13.7 efgh	806.5 ± 40.9 cde	14.7 ± 0.3 ab	9.3 ± 2.2 abcdef	6.6 ± 1.8 bcd	106.3 ± 6.7 cdef	16.6 ± 3.0 f	11.2 ± 2.1 abcd	45.4 ± 9.1 cdef
	Tun-Al-BV-5	44.1 ± 2.3 fgh	103.0 ± 11.7 gh	10.4 ± 0.8 bcdef	156.7 ± 8.3 fghi	809.2 ± 22.2 cde	1.7 ± 1.3 a	11.9 ± 0.6 abcdefg	7.7 ± 1.1 d	106.4 ± 8.6 cdef	1.5 ± 1.0 a	15.6 ± 0.8 cd	62.7 ± 4.2 g
	Tun-Al-CC-1	21.0 ± 4.4 abcd	53.0 ± 8.3 bcdef	5.6 ± 0.7 abcd	101.6 ± 9.5 de	737.5 ± 37.1 bcd	15.8 ± 1.2 ab	6.9 ± 9.4 abcd	4.1 ± 0.5 abcd	86.3 ± 5.4 bc	13.0 ± 1.1 bcdef	10.1 ± 1.0 abcd	39.5 ± 6.2 cde
	Tun-Ch-MU-3	38.4 ± 0.1 defg	76.7 ± 1.6 cdefg	7.9 ± 0.1 abcdef	129.9 ± 0.6 efg	804.2 ± 43.4 cde	17.1 ± 1.1 b	5.4 ± 0.2 ab	5.7 ± 0.2 bcd	103.8 ± 9.4 cdef	17.3 ± 0.2 f	15.0 ± 0.4 bcd	51.7 ± 2.4 fg
	Tup-Gy-AD-3-MBT	61.8 ± 9.0 hij	172.7 ± 21.5 jk	14.3 ± 3.0 ef	220.5 ± 20.9 jkl	1009.9 ± 57.7 fg	13.8 ± 3.8 ab	19.4 ± 2.1 g	12.6 ± 1.7 e	122.4 ± 6.4 fg	3.3 ± 1.4 ab	14.8 ± 1.4 bcd	47.2 ± 5.7 g
	Tup-Gy-AD-6-Pi	69.5 ± 4.3 j	147.5 ± 15.7 hij	12.7 ± 9.7 def	182.9 ± 20.0 hij	788.7 ± 29.5 cde	1.1 ± 1.6 a	15.2 ± 2.0 defg	7.3 ± 0.5 cd	94.3 ± 5.1 cde	5.6 ± 8.7 abcd	15.2 ± 0.8 bcd	42.6 ± 3.1 cde
	Tup-Gy-AD-6-Pr	92.8 ± 11.6 k	202.4 ± 35.9 k	5.4 ± 3.2 abcd	235.1 ± 23.4 kl	866.4 ± 42.3 de	3.8 ± 2.9 ab	19.8 ± 3.6 g	13.1 ± 3.8 e	89.2 ± 4.1 bcd	4.8 ± 3.7 abc	5.8 ± 8.4 abc	58.4 ± 8.5 fg
	Tup-Gy-AD-7	103.3 ± 2.9 k	210.7 ± 7.7 k	8.5 ± 2.1 abcdef	248.3 ± 3.7 l	908.0 ± 28.6 ef	4.8 ± 2.8 ab	20.1 ± 0.2 g	12.4 ± 1.0 e	96.1 ± 1.2 cde	6.3 ± 0.5 abcde	6.6 ± 10.1 abcd	64.5 ± 0.1 g
	Tup-Gy-AD-9	59.7 ± 3.4 hij	149.4 ± 12.1 ij	15.0 ± 1.7 f	196.9 ± 9.7 ijk	870.0 ± 19.1 de	8.6 ± 8.5 ab	16.6 ± 0.7 efg	6.9 ± 0.9 bcd	102.2 ± 3.7 cdef	16.5 ± 1.0 f	12.6 ± 0.6 abcd	35.7 ± 1.3 bcd
	Tup-Gy-GA-3	53.6 ± 9.4 ghij	167.0 ± 24.1 jk	8.5 ± 2.4 abcdef	222.9 ± 29.6 jkl	1084.8 ± 117.2 g	6.3 ± 9.5 ab	20.7 ± 3.9 g	16.4 ± 2.4 e	131.5 ± 17.0 g	6.0 ± 2.5 abcde	15.7 ± 2.5 cd	51.6 ± 7.7 efg
	Tup-SJ-JM-1	29.2 ± 1.9 cdef	80.0 ± 4.1 defg	7.6 ± 0.6 abcdef	124.5 ± 6.8 ef	795.1 ± 23.8 cde	14.7 ± 3.2 ab	5.1 ± 0.5 a	4.3 ± 0.2 abcd	103.2 ± 3.5 cdef	13.4 ± 0.5 cdef	9.7 ± 0.3 abcd	36.5 ± 2.7 bcd
	Lujan de Cuyo	11.8 ± 4.8 a	52.1 ± 25.8 ab	4.2 ± 1.8 abc	98.0 ± 32.8 abc	750.8 ± 37.8 b	9.8 ± 4.1 a	12.9 ± 4.6 ab	3.5 ± 0.6 a	101.2 ± 5.7 b	10.4 ± 2.0 a	8.6 ± 2.2 ab	41.7 ± 7.5 ab
	Maipu	9.4 ± 5.9 a	22.8 ± 13.8 ab	3.6 ± 0.9 ab	55.4 ± 19.2 ab	550.8 ± 105.3 a	12.3 ± 3.2 a	11.8 ± 4.0 ab	2.9 ± 0.3 a	78.2 ± 11.6 b	12.1 ± 0.7 a	8.7 ± 1.6 ab	43.5 ± 0.7 ab
Departments	Rivadavia	7.6 ± 2.0 a	8.6 ± 3.2 a	2.1 ± 0.8 a	22.6 ± 11.1 a	372.1 ± 17.3 a	4.2 ± 2.1 a	5.5 ± 0.7 a	1.6 ± 1.0 a	43.6 ± 6.3 a	6.5 ± 2.2 a	4.0 ± 1.2 a	25.6 ± 7.0 a
	San Carlos	43.3 ± 14.3 ab	94.6 ± 16.1 bc	9.4 ± 1.4 bc	147.7 ± 16.9 cd	817.5 ± 37.5 b	12.8 ± 1.3 a	9.5 ± 3.3 ab	4.9 ± 1.3 ab	103.3 ± 8.1 b	14.8 ± 1.8 a	12.7 ± 2.6 b	40.7 ± 5.1 ab
	Tunuyan	34.5 ± 12.0 ab	77.6 ± 25.0 ab	8.0 ± 2.4 abc	129.4 ± 27.5 bcd	783.6 ± 40.0 b	11.5 ± 8.5 a	8.1 ± 3.4 ab	5.8 ± 1.8 ab	98.8 ± 10.9 b	10.6 ± 8.2 a	13.6 ± 3.0 b	51.3 ± 11.6 b
	Tupungato	67.1 ± 24.8 b	161.4 ± 43.3 c	10.3 ± 3.7 c	204.4 ± 41.6 d	903.3 ± 109.3 b	7.6 ± 5.1 a	16.7 ± 5.5 b	10.4 ± 4.3 b	105.6 ± 15.6 b	8.0 ± 4.9 a	11.5 ± 4.2 ab	48.1 ± 10.9 b
	East zone	7.6 ± 2.0 a	8.6 ± 3.2 a	2.1 ± 0.8 a	22.6 ± 11.1 a	372.1 ± 17.3 a	4.2 ± 2.1 a	5.5 ± 0.7 a	1.6 ± 1.0 a	43.6 ± 6.3 a	6.5 ± 2.2 a	4.0 ± 1.2 a	25.6 ± 7.0 a
Zones	First zone	10.8 ± 4.7 a	40.4 ± 25.3 a	4.0 ± 1.4 a	80.9 ± 34.3 a	670.8 ± 124.5 b	10.8 ± 3.6 a	12.5 ± 3.9 a	3.2 ± 0.5 ab	92.0 ± 14.5 b	11.1 ± 1.7 a	8.6 ± 1.8 ab	42.4 ± 5.4 b
	Uco Valley	52.7 ± 23.4 b	122.3 ± 49.3 b	9.5 ± 2.8 b	170.5 ± 45.3 b	850.8 ± 92.1 c	10.1 ± 5.3 a	12.6 ± 5.8 a	7.7 ± 4.0 b	103.5 ± 12.1 b	10.8 ± 5.5 a	12.3 ± 3.4 b	46.3 ± 9.8 b

Location		LMW-PCs														
		Gallic acid - 370	OH-tyrosol - 82755	Tyrosol - 10393	(+)-Catechin - 9064	Syringic acid - 10742	(-)-Epicatechin - 72276	Astilbin - 119258	Caffeic acid - 689043	*p*-coumaric acid - 637542	Ferulic acid - 445858	*trans*-Piceid - 5281718	*trans*-resveratrol - 445154	Quercetin-3-glucoside - 5280804	Quercetin - 5280343	(-)-Gallocatechin - 9882981
	Luj-Agr-LP-1-80	30.8 ± 4.1 bcd	0.4 ± 0.6 a	19.1 ± 1.4 def	20.0 ± 7.7 a	4.2 ± 4.4 a	62.8 ± 1.5 ab	7.6 ± 2.3 a	14.0 ± 1.5 efg	19.7 ± 1.3 fghi	0.1 ± 0.1 ab	0.1 ± 0.0 a	2.5 ± 0.1 ab	4.3 ± 2.5 abc	9.3 ± 0.7 bcdefgh	5.8 ± 0.5 bcd
	Luj-Ug-FO-1	21.6 ± 3.3 abc	0.0 a	12.7 ± 1.9 ab	24.8 ± 9.1 a	11.3 ± 2.2 bcd	17.3 ± 6.6 ab	11.2 ± 2.9 abc	8.7 ± 1.0 cde	31.1 ± 5.1 jk	0.8 ± 0.1 defg	0.5 ± 0.4 a	8.9 ± 0.7 ef	2.5 ± 0.1 a	4.7 ± 0.7 ab	5.5 ± 0.7 bc
	Luj-Ug-ZA-1	29.7 ± 1.5 abcd	0.0 a	14.1 ± 0.4 abcd	27.6 ± 14.6 a	13.2 ± 2.8 bcdef	49.7 ± 34.3 ab	13.4 ± 2.8 abcd	11.7 ± 0.2 def	46.5 ± 2.4 l	0.6 ± 0.2 cdef	0.4 ± 0.1 a	7.9 ± 1.5 cdef	2.2 ± 1.3 a	5.6 ± 0.5 abc	8.4 ± 1.6 bcdef
	Mai-Lu-AG-18	25.1 ± 1.5 abcd	0.6 ± 0.5 abc	14.5 ± 0.9 abcde	22.2 ± 7.5 a	14.2 ± 0.5 bcdef	16.1 ± 7.2 ab	15.2 ± 4.4 abcd	6.4 ± 1.5 abcde	16.7 ± 3.5 efgh	0.8 ± 0.0 defg	0.9 ± 0.3 a	9.8 ± 1.6 ef	2.9 ± 0.8 ab	10.7 ± 2.8 cdefghi	6.2 ± 0.9 bcde
	Mai-Lu-AG-20	33.8 ± .1 bcd	0.9 ± 0.1 abc	17.7 ± 0.7 abcdef	27.9 ± 7.5 a	17.6 ± 0.7 fg	23.9 ± 4.2 ab	18.2 ± 1.8 abcd	6.0 ± 0.5 abcd	15.0 ± 2.2 efg	0.6 ± 0.0 cdef	1.0 ± 0.2 a	2.7 ± 0.3 ab	3.7 ± 0.4 abc	10.4 ± 1.0 cdefghi	8.0 ± 0.8 bcdef
	Riv-Lib-LL-1	37.6 ± 7.3 d	0.6 ± 1.0 ab	17.0 ± 1.2 abcdef	37.9 ± 12.8 a	14.6 ± 0.5 cdef	21.2 ± 5.7 ab	16.3 ± 1.8 abcd	21.7 ± 1.2 g	26.4 ± 3.9 ij	0.6 ± 0.3 cdef	0.4 ± 0.2 a	2.1 ± 0.0 ab	3.2 ± 0.3 ab	8.2 ± 1.0 abcde	8.4 ± 0.6 bcdef
	Riv-Mir-LV-4	54.1 ± 0.7 e	0.7 ± 0.7 abc	27.0 ± 0.7 h	26.5 ± 5.2 a	21.1 ± 3.2 g	57.1 ± 48.6 ab	19.6 ± 2.3 abcde	18.8 ± 4.2 fg	21.2 ± 2.3 ghi	0.02 + 0.03 a	0.1 ± 0.0 a	0.8 ± 0.3 a	3.0 ± 1.2 ab	14.8 ± 0.5 hij	10.2 ± 0.3 ef
	Riv-Riv-LA-1	15.1 ± 12.6 a	0.4 ± 0.6 a	18.8 ± 1.1 def	21.0 ± 16.1 a	15.8 ± 0.8 defg	12.4 ± 13.7 a	8.7 ± 4.7 ab	38.2 ± 9.1 h	38.9 ± 4.3 kl	0.03 ± 0.06 a	0.1 ± 0.1 a	0.2 ± 0.2 a	2.3 ± 1.0 a	3.5 ± 0.5 a	1.1 ± 1.8 a
	San-Alt-NI-1-Pi	31.9 ± 1.4 bcd	3.5 ± 2.3 abcde	20.6 ± 0.7 fg	24.1 ± 5.4 a	15.1 ± 0.5 cdef	18.8 ± 6.9 ab	13.7 ± 1.9 abcd	7.8 ± 1.8 abcde	25.9 ± 2.4 ij	0.9 ± 0.1 defg	0.1 ± 0.0 a	6.8 ± 1.5 cde	2.1 ± 0.8 a	6.6 ± 0.8 abc	9.1 ± 0.3 bcdef
	San-Alt-NI-1-Pr	28.4 ± 2.2 abcd	5.4 ± 0.8 bcde	19.7 ± 0.7 def	21.9 ± 9.4 a	14.6 ± 0.3 cdef	16.6 ± 7.0 ab	16.0 ± 2.0 abcd	7.1 ± 0.8 abcde	24.9 ± 3.0 hij	0.9 ± 0.1 efg	0.1 ± 0.0 a	8.1 ± 0.8 def	1.8 ± 0.4 a	7.3 ± 0.7 abcd	8.2 ± 1.1 bcdef
	San-Alt-NI-2-Pr	28.4 ± 1.4 abcd	3.2 ± 0.6 abcd	18.0 ± 1.1 bcdef	54.1 ± 11.9 a	11.8 ± 1.4 bcde	29.2 ± 2.2 ab	19.0 ± 1.2 abcde	7.9 ± 0.5 bcde	26.1 ± 1.1 ij	1.1 ± 0.1 g	0.5 ± 0.2 a	11.1 ± 1.2 f	3.2 ± 0.6 ab	8.4 ± 0.8 abcdef	10.1 ± 0.5 ef
Parcels	San-Cep-EB-3	26.2 ± 8.4 abcd	1.5 ± 1.5 abcd	15.6 ± 3.8 abcdef	25.4 ± 17.5 a	10.2 ± 1.7 bc	24.1 ± 23.1 ab	13.7 ± 7.1 abcd	5.4 ± 0.8 abcd	19.7 ± 3.8 fghi	0.5 ± 0.3 bcd	0.4 ± 0.2 a	2.5 ± 0.7 ab	2.0 ± 0.5 a	8.6 ± 1.5 abcdefg	8.0 ± 1.9 bcdef
	San-Cep-EC-5	25.7 ± 0.1 abcd	8.2 ± 1.8 e	18.5 ± 1.5 bcdef	32.5 ± 11.8 a	10.7 ± 1.0 bcd	33.3 ± 10.1 ab	16.4 ± 1.8 abcd	3.0 ± 0.1 abc	11.2 ± 0.7 cde	0.8 ± 0.1 defg	1.1 ± 0.4 a	3.0 ± 1.2 ab	2.9 ± 0.2 ab	7.1 ± 1.3 abc	6.1 ± 1.0 bcde
	Tun-Al-BV-5	35.6 ± 2.8 cd	6.0 ± 1.0 de	26.0 ± 2.2 gh	28.6 ± 6.9 a	17.2 ± 1.8 efg	36.4 ± 9.7 ab	19.4 ± 3.3 abcde	0.6 ± 0.1 ab	3.7 ± 0.3 abc	0.5 ± 0.3 cde	1.4 ± 0.4 a	1.1 ± 0.3 a	4.8 ± 1.2 abc	13.7 ± 0.6 fghij	7.7 ± 1.3 bcdef
	Tun-Al-CC-1	30.2 ± 1.7 abcd	4.4 ± 0.5 abcde	18.6 ± 4.3 bcdef	32.8 ± 9.8 a	15.7 ± 1.6 defg	24.5 ± 7.8 ab	11.8 ± 1.0 abcd	8.0 ± 1.6 bcde	24.3 ± 1.9 hij	0.7 ± 0.1 defg	0.3 ± 0.2 a	9.8 ± 1.0 ef	4.1 ± 1.8 abc	11.1 ± 2.6 cdefghi	9.3 ± 0.7 cdef
	Tun-Ch-MU-3	27.0 ± 3.7 abcd	6.1 ± 5.3 de	20.2 ± 0.6 efg	27.3 ± 17.5 a	14.8 ± 1.0 cdef	32.6 ± 8.9 ab	21.1 ± 3.8 bcde	2.2 ± 1.1 abc	12.5 ± 2.3 def	0.8 ± 0.1 defg	0.7 ± 0.2 a	7.6 ± 1.6 cde	3.1 ± 1.0 ab	14.0 ± 1.6 ghij	8.0 ± 0.6 bcdef
	Tup-Gy-AD-3-MBT	22.5 ± 4.2 abcd	1.0 ± 1.8 abc	13.9 ± 1.0 abcd	29.6 ± 22.1 a	10.6 ± 0.6 bcd	28.5 ± 14.9 ab	24.6 ± 6.6 defg	0.9 ± 0.2 ab	5.9 ± 1.7 abcd	0.7 ± 0.1 cdefg	1.0 ± 0.5 a	4.7 ± 2.1 bc	4.7 ± 1.3 abc	18.2 ± 2.9 jk	8.5 ± 1.8 bcdef
	Tup-Gy-AD-6-Pi	31.3 ± 6.8 bcd	4.3 ± 0.4 abcde	18.8 ± 2.8 cdef	56.9 ± 8.8 a	12.4 ± 1.6 bcdef	68.6 ± 5.5 b	35.5 ± 1.8 fg	0.2 ± 0.2 a	4.4 ± 1.5 abcd	0.5 ± 0.2 bcde	0.1 ± 0.2 a	3.3 ± 0.4 ab	11.4 ± 2.5 d	20.5 ± 2.0 kl	10.0 ± .2 ef
	Tup-Gy-AD-6-Pr	25.1 ± 2.3 abcd	3.5 ± 0.2 abcde	18.2 ± 2.7 bcdef	43.2 ± 6.4 a	12.4 ± 3.3 bcdef	44.1 ± 3.3 ab	32.1 ± 2.9 efg	0.5 ± 0.1 ab	1.7 ± 0.4 a	0.9 ± 0.1 defg	3.8 ± 1.8 b	1.9 ± 1.0 ab	6.4 ± 0.4 bc	13.7 ± 3.2 efghij	9.0 ± 1.1 bcdef
	Tup-Gy-AD-7	27.3 ± 1.6 abcd	3.9 ± 0.9 abcde	18.1 ± 1.7 bcdef	55.6 ± 9.0 a	12.5 ± 1.0 bcdef	46.4 ± 6.3 ab	37.0 ± 3.8 g	0.6 ± 0.1 ab	2.4 ± 1.1 ab	1.0 ± 0 fg	3.3 ± 0.7 b	1.5 ± 0.6 ab	7.0 ± 0.9 c	24.8 ± 2.0 l	11.1 ± 1.2 f
	Tup-Gy-AD-9	26.2 ± 4.3 abcd	2.4 ± 1.4 abcd	17.5 ± 1.0 abcdef	35.3 ± 28.6 a	12.2 ± 0.8 bcdef	30.3 ± 17.5 ab	22.9 ± 6.3 cdef	0.9 ± 0.2 ab	4.4 ± 1.4 abcd	0.7 ± 0.1 defg	0.7 ± 0.3 a	4.9 ± 0.5 bcd	4.4 ± 0.9 abc	12.8 ± 2.0 defghij	9.6 ± 1.7 def
	Tup-Gy-GA-3	18.8 ± 3.8 ab	3.2 ± 3.7 abcd	11.8 ± 0.8 a	37.3 ± 43.7 a	9.0 ± 0.7 ab	34.2 ± 31.6 ab	16.5 ± 9.1 abcd	2.3 ± 0.6 abc	10.3 ± 1.3 bcde	0.6 ± 0.1 cdef	1.1 ± 0.2 a	19.7 ± 2.2 g	2.5 ± 0.6 a	15.8 ± 2.0 ijk	9.9 ± 3.0 def
	Tup-SJ-JM-1	18.7 ± 1.2 ab	5.5 ± 0.6 cde	12.8 ± 0.5 abc	13.3 ± 5.4 a	9.7 ± 0.3 bc	19.4 ± 6.7 ab	19.6 ± 3.1 abcde	0.6 ± 0.2 ab	3.4 ± 0.2 abc	0.3 ± 0.1 abc	1.2 ± 0.3 a	1.9 ± 0.2 ab	3.4 ± 0.9 ab	9.3 ± 0.5 bcdefgh	5.1 ± 0.2 ab
	Lujan de Cuyo	27.4 ± 5.0 a	0.1 ± 0.2 a	15.3 ± 3.3 a	24.1 ± 3.9 a	9.5 ± 4.7 a	43.2 ± 23.4 a	10.7 ± 2.9 a	11.4 ± 2.6 b	32.4 ± 13.4 c	0.5 ± 0.4 a	0.3 ± 0.2 a	6.4 ± 3.4 a	3.0 ± 1.1 a	6.5 ± 2.5 a	6.6 ± 1.6 a
	Maipu	29.5 ± 6.2 a	0.7 ± 0.2 ab	16.1 ± 2.3 a	25.1 ± 4.1 a	15.9 ± 2.4 ab	20.0 ± 5.5 a	16.7 ± 2.1 ab	6.2 ± 0.3 ab	15.9 ± 1.2 abc	0.7 ± 0.1 a	1.0 ± 0.1 a	6.2 ± 5.0 a	3.3 ± 0.5 a	10.6 ± 0.2 ab	7.1 ± 1.3 a
Departments	Rivadavia	35.6 ± 19.6 a	0.6 ± 0.2 ab	20.9 ± 5.3 a	28.5 ± 8.6 a	17.2 ± 3.4 b	30.2 ± 23.7 a	14.9 ± 5.6 ab	26.2 ± 10.5 c	28.8 ± 9.1 bc	0.2 ± 0.3 a	0.2 ± 0.2 a	1.0 ± 1.0 a	2.8 ± 0.5 a	8.8 ± 5.7 ab	6.6 ± 4.8 a
	San Carlos	28.1 ± 2.4 a	4.4 ± 2.5 bc	18.5 ± 1.9 a	31.6 ± 13.2 a	12.5 ± 2.3 ab	24.4 ± 7.0 a	15.8 ± 2.2 ab	6.2 ± 2.1 ab	21.6 ± 6.4 abc	0.8 ± 0.2 a	0.4 ± 0.4 a	6.3 ± 3.6 a	2.4 ± 0.6 a	7.6 ± 0.9 ab	8.3 ± 1.5 a
	Tunuyan	30.9 ± 4.3 a	5.5 ± 1.0 c	21.6 ± 3.9 a	29.6 ± 2.9 a	15.9 ± 1.2 b	31.2 ± 6.1 a	17.4 ± 4.9 ab	3.6 ± 3.9 ab	13.5 ± 10.3 ab	0.7 ± 0.1 a	0.8 ± 0.5 a	6.2 ± 4.5 a	4.0 ± 0.8 a	12.9 ± 1.6 ab	8.3 ± 0.9 a
	Tupungato	24.3 ± 4.6 a	3.4 ± 1.4 abc	15.9 ± 2.9 a	38.8 ± 15.2 a	11.3 ± 1.5 ab	38.8 ± 16.1 a	26.9 ± 8.0 b	0.9 ± 0.7 a	4.6 ± 2.9 a	0.7 ± 0.2 a	1.6 ± 1.4 a	5.4 ± 6.4 a	5.7 ± 3.0 a	16.4 ± 5.2 b	9.0 ± 1.9 a
	East zone	35.6 ± 19.6 a	0.6 ± 0.2 ab	20.9 ± 5.3 a	28.5 ± 8.6 a	17.2 ± 3.4 a	30.2 ± 23.7 a	14.9 ± 5.6 a	26.2 ± 10.5 b	28.8 ± 9.1 b	0.2 ± 0.3 a	0.2 ± 0.2 a	1.0 ± 1.0 a	2.8 ± 0.5 a	8.8 ± 5.7 a	6.6 ± 4.8 a
Zones	First zone	28.2 ± 4.8 a	0.4 ± 0.4 a	15.6 ± 2.7 a	24.5 ± 3.4 a	12.1 ± 5.0 a	33.9 ± 21.1 a	13.1 ± 4.0 a	9.3 ± 3.4 a	25.8 ± 13.1 ab	0.6 ± 0.3 ab	0.6 ± 0.4 a	6.3 ± 3.5 a	3.1 ± 0.9 a	8.2 ± 2.8 a	6.8 ± 1.3 a
	Uco Valley	26.9 ± 4.6 a	4.1 ± 1.9 b	17.9 ± 3.5 a	34.5 ± 12.9 a	12.6 ± 2.4 a	32.5 ± 13.2 a	21.3 ± 7.9 a	3.2 ± 3.1 a	12.0 ± 9.5 a	0.7 ± 0.2 b	1.0 ± 1.1 a	5.9 ± 5.0 a	4.3 ± 2.5 a	12.8 ± 5.3 a	8.7 ± 1.6 a

Different letters within the same row indicate significant differences ($p < 0.05$) according to a Tukey HSD test.

Table S3. Overall data of anthocyanins and LMW-PCs (Name and PubChem CID) quantified [Mean (mg L−1) ± SD] in Malbec wines from different locations of Mendoza obtained in 2018 vintage.

	Location	Anthocyanins											
		Delphinidin 3-O-glucoside - 102515359	Cyanidin 3-O-glucoside - 197081	Petunidin 3-O-glucoside - 443651	Peonidin 3-O-glucoside - 443654	Malvidin 3-O-glucoside - 443652	Delphinidin 3-O-acetylglucoside - 15385440	Petunidin 3-O-acetylglucoside - 44256961	Peonidin 3-O-acetylglucoside - 44256847	Malvidin 3-O-acetylglucoside - 72193646	Petunidin 3-O-*p*-coumaroylglucoside - 9897848	Peonidin 3-O-*p*-coumaroylglucoside - 131752297	Malvidin 3-O-*p*-coumaroylglucoside - 71308234
Parcels	Luj-Agr-LP-1-80	13.7 ± 2.7 abc	81.8 ± 3.3 defg	0.4 ± 0.1 cdef	138.5 ± 1.3 efgh	843.2 ± 38.8 defg	11.6 ± 8.9 a	10.8 ± 0.9 abcd	3.5 ± 0.3 cd	133.5 ± 10.2 defg	1.3 ± 0.5 ab	11.3 ± 0.9 cdefg	65.6 ± 5.8 cdef
	Luj-Ug-FO-1	12.1 ± 8.5 abc	9.7 ± 3.6 a	0.1 ± 0.1 abcd	39.3 ± 6.5 abc	573.5 ± 80.8 abc	7.8 ± 3.1 a	9.8 ± 1.3 abcd	3.3 ± 1.4 bcd	80.7 ± 16.8 ab	7.6 ± 1.3 abc	6.0 ± 1.1 ab	38.9 ± 7.3 ab
	Luj-Ug-ZA-1	13.5 ± 0.1 abc	5.1 ± 2.1 a	0.2 ± 0.0 abcdef	31.2 ± 2.5 abc	498.9 ± 5.6 ab	12.4 ± 3.5 a	7.7 ± 0.0 ab	2.4 ± 0.6 abc	70.9 ± 0.4 a	6.8 ± 0.4 abc	3.6 ± 0.1 a	38.7 ± 1.1 ab
	Mai-Lu-AG-18	10.4 ± 9.0 abc	32.6 ± 14.8 abc	0.2 ± 0.1 abcdef	69.7 ± 23.3 bcd	617.9 ± 148.4 abc	16.4 ± 2.4 a	9.7 ± 8.2 abcd	3.3 ± 0.4 bcd	90.7 ± 19.9 abc	12.2 ± 3.0 abc	9.5 ± 2.9 bcd	44.7 ± 14.7 abc
	Mai-Lu-AG-20	13.3 ± 3.0 abc	33.1 ± 0.9 abc	0.2 ± 0.2 abcdef	73.5 ± 4.2 cd	684.8 ± 47.4 bcd	8.6 ± 5.5 a	16.6 ± 0.7 def	3.2 ± 1.3 bcd	111.3 ± 12.3 bcd	14.5 ± 0.4 bc	12.5 ± 0.9 cdefghi	57.6 ± 2.8 bcdef
	Riv-Lib-LL-1	9.8 ± 0.9 ab	8.2 ± 9.0 a	0.04 ± 0.0 ab	26.6 ± 1.1 ab	459.8 ± 18.5 a	5.1 ± 1.8 a	6.4 ± 0.5 a	1.3 ± 0.3 ab	62.5 ± 2.5 a	8.1 ± 0.5 abc	3.5 ± 0.3 a	45.8 ± 4.8 abcd
	Riv-Mir-LV-4	5.6 ± 2.9 a	10.9 ± 0.6 a	0.1 ± 0.0 abc	39.9 ± 2.8 abc	493.1 ± 24.8 ab	6.8 ± 10.1 a	8.2 ± 0.5 abc	4.8 ± 0.1 de	58.1 ± 3.2 a	10.7 ± 0.7 abc	8.5 ± 1.1 bc	59.2 ± 5.0 bcdef
	Riv-Riv-LA-1	6.7 ± 1.3 a	14.1 ± 2.1 ab	0.01 ± 0.0 a	17.1 ± 5.6 a	463.8 ± 67.3 a	3.7 ± 1.2 a	4.9 ± 0.9 a	0.7 ± 0.2 a	57.6 ± 11.1 a	4.1 ± 0.8 abc	1.8 ± 0.4 a	25.9 ± 6.2 a
	San-Alt-NI-1-Pi	33.6 ± 6.2 defgh	145.0 ± 12.9 ij	0.5 ± 0.1 f	202.3 ± 15.3 k	1119.9 ± 87.6 h	10.4 ± 4.2 a	23.0 ± 2.5 f	5.1 ± 0.6 de	182.1 ± 16.9 h	4.5 ± 1.4 abc	12.7 ± 0.8 cdefghi	68.6 ± 5.8 ef
	San-Alt-NI-1-Pr	16.0 ± 2.1 abcd	66.0 ± 11.3 cde	0.3 ± 0.0 abcdef	120.7 ± 10.7 ef	969 ± 44.3 fgh	11.1 ± 9.5 a	7.8 ± 2.3 ab	2.7 ± 0.3 abc	157.7 ± 8.6 gh	15.9 ± 0.6 c	10.6 ± 0.7 cdef	53.9 ± 5.0 bcde
	San-Alt-NI-2-Pr	28.5 ± 3.7 cdefg	118.4 ± 9.6 ghi	0.4 ± 0.2 bcdef	180.1 ± 16.6 hijk	1039.4 ± 86.1 gh	7.6 ± 4.7 a	18.8 ± 2.3 ef	4.2 ± 0.8 cde	178.5 ± 22.3 h	1.7 ± 1.6 ab	12.2 ± 1.0 cdefgh	59.6 ± 6.4 bcdef
	San-Cep-EB-3	20.5 ± 1.4 abcde	94.6 ± 6.9 efgh	0.3 ± 0.1 abcdef	151.1 ± 5.8 efghi	919.5 ± 31.3 efgh	2.2 ± 1.3 a	10.0 ± 0.7 abcd	3.7 ± 0.5 cde	133.3 ± 7.6 defg	0.3 ± 0.1 a	9.6 ± 0.2 bcde	56.0 ± 4.2 bcdef
	San-Cep-EC-5	15.2 ± 0.9 abc	96.0 ± 7.3 efgh	0.5 ± 0.2 f	156.5 ± 6.4 fghij	911.0 ± 45.6 efg	1.4 ± 1.0 a	16.9 ± 1.8 def	3.7 ± 0.4 cde	148.3 ± 13.7 efgh	6.9 ± 9.8 abc	11.2 ± 1.4 cdef	54.7 ± 6.0 bcde
	Tun-Al-BV-5	26.7 ± 3.4 bcdef	97.6 ± 12.2 efgh	0.5 ± 0.2 def	154.8 ± 18.0 fghij	983.9 ± 62.4 fgh	12.0 ± 1.2 a	12.0 ± 2.0 abcde	5.2 ± 0.3 de	148.6 ± 7.4 fgh	12.6 ± 9.4 abc	13.8 ± 1.9 efghi	65.5 ± 4.4 cdef
	Tun-Al-CC-1	37.9 ± 7.6 efgh	111.6 ± 5.9 fghi	0.5 ± 0.1 f	165.1 ± 4.0 fghijk	907.6 ± 22.0 efg	8.5 ± 3.4 a	11.6 ± 0.7 abcde	5.6 ± 1.0 e	124.6 ± 2.6 cdefg	1.7 ± 0.5 ab	15.7 ± 2.2 hi	61.3 ± 4.7 cdef
	Tun-Ch-MU-3	4.5 ± 1.0 a	51.1 ± 3.4 bcd	0.5 ± 0.1 ef	107.7 ± 6.3 de	831.4 ± 50.4 def	14.2 ± 2.5 a	6.6 ± 0.7 a	2.7 ± 0.3 abc	133.8 ± 8.3 defg	12.4 ± 10.1 abc	11.9 ± 1.0 cdefgh	63.0 ± 8.1 cdef
	Tup-Gy-AD-3-MBT	40.8 ± 5.1 fgh	133.2 ± 3.0 hij	0.2 ± 0.1 abcdef	156.6 ± 13.8 fghij	736.2 ± 78.9 cde	3.4 ± 2.3 a	10.9 ± 3.1 abcd	4.3 ± 0.4 cde	92.4 ± 16.4 abc	7.4 ± 9.8 abc	12.9 ± 2.1 defghi	56.0 ± 10.5 bcdef
	Tup-Gy-AD-6-Pi	37.6 ± 1.7 efgh	117.2 ± 6.7 fghi	0.3 ± 0.0 abcdef	167.3 ± 2.0 ghijk	860.0 ± 23.4 defg	1.7 ± 1.9 a	14.6 ± 0.6 bcde	3.4 ± 0.7 cd	114.5 ± 3.7 bcdef	2.0 ± 0.2 ab	13.8 ± 0.7 defghi	59.5 ± 2.2 bcdef
	Tup-Gy-AD-6-Pr	47.9 ± 13.7 h	163.0 ± 34.2 j	0.4 ± 0.2 abcdef	199.7 ± 33.0 jk	849.9 ± 75.7 defg	7.6 ± 2.8 a	17.0 ± 2.6 def	5.1 ± 0.2 de	110.3 ± 7.7 bcd	4.5 ± 2.5 abc	15.8 ± 1.9 hi	58.0 ± 8.2 bcdef
	Tup-Gy-AD-7	45.0 ± 9.2 gh	140.0 ± 18.5 ij	0.4 ± 0.1 bcdef	185.5 ± 21.8 ijk	877.8 ± 40.4 defg	8.3 ± 8.1 a	16.4 ± 2.6 def	3.5 ± 1.1 cd	111.9 ± 0.7 bcde	4.2 ± 2.5 abc	15.5 ± 1.5 ghi	67.3 ± 11.8 def
	Tup-Gy-AD-9	34.7 ± 4.8 efgh	131.6 ± 11.1 hij	0.4 ± 0.1 abcdef	173.8 ± 18.5 ghijk	899.6 ± 91.3 efg	7.5 ± 5.1 a	15.5 ± 1.9 bcdef	5.0 ± 0.7 de	118.6 ± 12.8 cdef	2.0 ± 1.9 ab	14.7 ± 1.6 fghi	56.5 ± 9.1 bcdef
	Tup-Gy-GA-3	47.8 ± 11.9 h	148.7 ± 29.4 ij	0.3 ± 0.1 abcdef	183.0 ± 30.5 hijk	895.6 ± 90.3 efg	10.5 ± 9.2 a	16.0 ± 4.6 cdef	3.4 ± 0.5 cd	123.6 ± 16.9 cdefg	6.7 ± 3.7 abc	16.7 ± 1.9 i	77.9 ± 10.5 f
	Tup-SJ-JM-1	25.2 ± 1.1 bcdef	78.3 ± 2.1 def	0.1 ± 0.1 abcde	130.7 ± 1.8 efg	933.5 ± 11.7 efgh	15.1 ± 3.2 a	6.5 ± 0.5 a	2.5 ± 0.4 abc	132.4 ± 6.3 defg	16.0 ± 0.8 c	11.2 ± 0.4 cdefg	58.0 ± 1.6 bcdef
Departments	Lujan de Cuyo	13.1 ± 0.9 a	32.2 ± 43.0 ab	0.3 ± 0.2 abc	69.7 ± 59.8 ab	638.5 ± 181.1 a	10.6 ± 2.5 a	9.5 ± 1.6 a	3.1 ± 0.6 a	95.0 ± 33.7 ab	5.2 ± 3.4 a	7.0 ± 3.9 ab	47.7 ± 15.5 a
	Maipu	11.9 ± 2.0 a	32.9 ± 0.3 ab	0.2 ± 0.0 ab	71.6 ± 2.7 ab	651.4 ± 47.3 ab	12.5 ± 5.5 a	13.1 ± 4.9 a	3.3 ± 0.1 a	101.0 ± 14.6 ab	13.3 ± 1.7 a	11.0 ± 2.1 bc	51.1 ± 9.1 a
	Rivadavia	7.4 ± 2.2 a	11.0 ± 3.0 a	0.1 ± 0.0 a	27.9 ± 11.4 a	472.3 ± 18.2 a	5.2 ± 1.6 a	6.5 ± 1.6 a	2.3 ± 2.2 a	59.4 ± 2.7 a	7.7 ± 3.3 a	4.6 ± 3.5 a	43.6 ± 16.7 a
	San Carlos	22.8 ± 8.0 ab	104.0 ± 29.5 c	0.4 ± 0.1 bc	162.1 ± 30.8 c	991.8 ± 88.0 c	6.5 ± 4.5 a	15.3 ± 6.3 a	3.9 ± 0.9 a	160.0 ± 20.5 c	5.9 ± 6.2 a	11.3 ± 1.3 bc	58.6 ± 6.0 a
	Tunuyan	23 ± 17.0 ab	86.8 ± 31.6 bc	0.5 ± 0.0 c	142.5 ± 30.6 bc	907.7 ± 76.3 c	11.6 ± 2.9 a	10.0 ± 3.0 a	4.5 ± 1.6 a	135.7 ± 12.1 bc	8.9 ± 6.2 a	13.8 ± 1.9 c	63.3 ± 2.1 a
	Tupungato	39.9 ± 8.2 b	130.3 ± 27.0 c	0.3 ± 0.1 bc	170.9 ± 22.5 c	864.7 ± 63.0 bc	7.7 ± 4.4 a	13.9 ± 3.8 a	3.9 ± 1.0 a	114.8 ± 12.5 b	6.1 ± 4.9 a	14.4 ± 1.9 c	61.9 ± 8.0 a
Zones	East zone	7.4 ± 2.2 a	11.0 ± 3.0 a	0.1 ± 0.0 a	27.9 ± 11.4 a	472.3 ± 18.2 a	5.2 ± 1.6 a	6.5 ± 1.6 a	2.3 ± 2.2 a	59.4 ± 2.7 a	7.7 ± 3.3 a	4.6 ± 3.5 a	43.6 ± 16.7 a
	First zone	12.6 ± 1.4 a	32.5 ± 30.4 a	0.2 ± 0.1 ab	70.4 ± 42.3 a	643.7 ± 130.4 b	11.4 ± 3.4 a	10.9 ± 3.4 ab	3.1 ± 0.4 a	97.4 ± 25.1 ab	8.5 ± 5.1 a	8.6 ± 3.7 a	49.1 ± 12.0 ab
	Uco Valley	30.8 ± 12.9 b	112.8 ± 32.1 b	0.4 ± 0.1 bc	162.3 ± 27.3 b	915.6 ± 90.1 c	8.1 ± 4.3 a	13.6 ± 4.8 b	4.0 ± 1.0 a	134.0 ± 25.2 b	6.6 ± 5.3 a	13.2 ± 2.2 b	61.1 ± 6.5 b

	Location	LMW-PCs														
		Gallic acid - 370	OH-tyrosol - 82755	Tyrosol - 10393	(+)-Catechin - 9064	Syringic acid - 10742	(-)-Epicatechin - 72276	Astilbin - 119258	Caffeic acid - 689043	*p*-coumaric acid - 637542	Ferulic acid - 445858	*trans*-Piceid - 5281718	*trans*-resveratrol - 445154	Quercetin-3-glucoside - 5280804	Quercetin - 5280343	(-)-Gallocatechin - 9882981
Parcels	Luj-Agr-LP-1-80	19.6 ± 0.7 cdef	1.1 ± 0.2 a	30.2 ± 3.6 e	3.3 ± 1.0 ab	6.9 ± 1.1 g	79.0 ± 6.7 bcd	15.0 ± 4.8 a	8.8 ± 2.3 ab	18.2 ± 2.2 defghi	0.7 ± 0.0 a	0.7 ± 0.2 a	1.6 ± 1.1 ab	1.5 ± 1.0 abc	11.6 ± 2.9 cde	5.2 ± 0.6 ab
	Luj-Ug-FO-1	16.0 ± 4.0 abcdef	0.9 ± 0.6 a	6.3 ± 1.0 abc	1.4 ± 1.0 ab	4.3 ± 0.5 abcde	60.8 ± 9.7 abc	20.7 ± 13.8 a	11.1 ± 9.5 ab	24.0 ± 5.5 ghi	0.6 ± 0.1 a	0.5 ± 0.3 a	3.6 ± 0.8 ab	3.8 ± 1.3 bc	4.2 ± 0.6 a	2.8 ± 1.5 ab
	Luj-Ug-ZA-1	14.9 ± 0.7 abcd	0.6 ± 0.1 a	15.9 ± 5.0 abcde	1.6 ± 1.4 ab	3.8 ± 0.9 abcd	135.6 ± 47.6 e	17.5 ± 6.8 a	6.5 ± 2.7 ab	29.2 ± 9.6 i	0.5 ± 0.2 a	0.6 ± 0.2 a	0.7 ± 0.6 a	1.8 ± 1.9 abc	3.7 ± 0.5 a	4.7 ± 1.2 ab
	Mai-Lu-AG-18	18.1 ± 0.8 bcdef	0.9 ± 0.2 a	6.5 ± 1.2 abc	1.4 ± 1.1 ab	5.4 ± 0.4 cdefg	60.5 ± 5.6 abc	25.8 ± 4.1 a	3.7 ± 1.5 a	8.7 ± 3.3 abcdef	0.3 ± 0.0 a	1.5 ± 0.3 ab	2.7 ± 1.3 ab	0.4 ± 0.2 ab	8.3 ± 1.0 abcd	4.6 ± 1.3 ab
	Mai-Lu-AG-20	11.5 ± 0.3 ab	0.9 ± 0.3 a	3.4 ± 1.8 a	1.7 ± 1.0 ab	2.7 ± 0.7 ab	44.7 ± 15.4 abc	19.7 ± 6.0 a	2.7 ± 1.9 a	3.5 ± 1.7 ab	0.4 ± 0.2 a	1.8 ± 0.7 ab	1.1 ± 0.3 a	2.0 ± 1.0 abc	7.0 ± 1.8 abcd	4.3 ± 0.6 ab
	Riv-Lib-LL-1	17.8 ± 1.9 bcdef	0.5 ± 0.1 a	6.2 ± 2.3 abc	2.1 ± 1.9 ab	4.5 ± 1.1 bcdefg	58.0 ± 15.3 abc	12.3 ± 2.2 a	26.4 ± 19.8 b	24.2 ± 8.1 hi	0.6 ± 0.6 a	0.5 ± 0.1 a	0.4 ± 0.2 a	2.0 ± 0.9 abc	4.2 ± 0.9 a	4.9 ± 2.1 ab
	Riv-Mir-LV-4	22.8 ± 2.3 f	1.2 ± 0.3 a	8.6 ± 0.5 abc	1.5 ± 0.6 ab	5.6 ± 0.3 defg	91.7 ± 6.2 cde	31.3 ± 8.6 a	16.0 ± 2.8 ab	31.3 ± 2.0 i	0.9 ± 0.0 a	0.9 ± 0.2 a	5.7 ± 1.0 bc	1.1 ± 1.1 abc	9.5 ± 0.9 abcd	6.0 ± 0.4 b
	Riv-Riv-LA-1	17.1 ± 2.4 bcdef	0.5 ± 0.1 a	13.8 ± 2.7 abcd	0.0 a	4.5 ± 0.5 abcdefg	119.0 ± 27.8 de	14.1 ± 3.6 a	59.7 ± 10.5 c	45.0 ± 10.4 j	2.7 ± 0.8 b	0.8 ± 0.1 a	1.0 ± 0.4 a	1.9 ± 0.7 abc	4.6 ± 0.7 ab	3.7 ± 0.7 ab
	San-Alt-NI-1-Pi	14.9 ± 5.0 abcd	0.8 ± 0.5 a	16.4 ± 3.1 abcde	1.6 ± 1.0 ab	5.6 ± 0.6 defg	51.3 ± 3.2 abc	23.3 ± 14.9 a	4.2 ± 0.9 a	15.2 ± 1.2 bcdefgh	0.6 ± 0.0 a	0.4 ± 0.3 a	1.3 ± 0.7 a	1.5 ± 0.9 abc	7.8 ± 1.8 abcd	3.1 ± 0.3 ab
	San-Alt-NI-1-Pr	15.2 ± 1.5 abcde	1.1 ± 0.3 a	9.8 ± 3.9 abcd	1.2 ± 1.1 a	4.3 ± 0.9 abcde	62.2 ± 6.1 abc	27.3 ± 9.2 a	5.8 ± 1.1 a	21.6 ± 4.4 fghi	0.6 ± 0.1 a	0.4 ± 0.1 a	3.2 ± 1.0 ab	3.5 ± 2.8 abc	6.6 ± 1.0 abc	3.4 ± 1.2 ab
	San-Alt-NI-2-Pr	17.4 ± 1.1 bcdef	1.2 ± 0.3 ab	18.7 ± 8.2 cde	2.4 ± 2.3 ab	6.8 ± 1.2 fg	64.0 ± 13.6 abc	32.6 ± 3.4 a	5.4 ± 1.5 a	18.2 ± 4.4 defghi	0.8 ± 0.1 a	0.7 ± 0.1 a	1.6 ± 0.5 ab	3.9 ± 2.9 bc	10.0 ± 2.2 abcde	3.9 ± 0.6 ab
	San-Cep-EB-3	16.5 ± 2.1 abcdef	1.1 ± 0.6 a	5.6 ± 0.5 abc	2.8 ± 0.9 ab	5.5 ± 1.0 defg	47.7 ± 5.6 abc	22.0 ± 9.8 a	10.3 ± 6.3 ab	17.9 ± 3.6 cdefghi	0.5 ± 0.1 a	0.5 ± 0.2 a	0.8 ± 0.3 a	1.4 ± 0.4 abc	9.2 ± 2.5 abcd	2.9 ± 0.8 ab
	San-Cep-EC-5	22.2 ± 1.5 ef	1.2 ± 0.5 a	23.5 ± 1.7 de	2.1 ± 0.6 ab	6.6 ± 0.5 efg	64.6 ± 8.6 abc	30.8 ± 8.2 a	8.1 ± 1.2 ab	14.0 ± 1.2 abcdefgh	0.6 ± 0.1 a	0.6 ± 0.1 a	1.2 ± 0.2 a	0.8 ± 0.2 ab	7.9 ± 1.0 abcd	4.0 ± 0.9 ab
	Tun-Al-BV-5	14.3 ± 1.2 abcd	0.9 ± 0.2 a	3.8 ± 1.5 ab	1.5 ± 0.6 ab	3.6 ± 0.9 abcd	48.3 ± 14.3 abc	15.6 ± 5.2 a	2.7 ± 0.7 a	6.5 ± 1.2 abcde	0.3 ± 0.1 a	0.6 ± 0.1 a	2.0 ± 1.2 ab	1.3 ± 0.3 abc	7.8 ± 3.0 abcd	4.0 ± 1.3 ab
	Tun-Al-CC-1	19.9 ± 0.4 def	3.1 ± 0.8 b	17.9 ± 12.5 bcde	1.3 ± 0.8 a	5.3 ± 0.3 cdefg	88.7 ± 13.2 cde	32.0 ± 4.6 a	15.7 ± 4.4 ab	19.5 ± 1.8 efghi	0.6 ± 0.2 a	1.7 ± 0.5 ab	9.1 ± 4.8 c	4.8 ± 0.8 c	18.4 ± 4.0 f	5.2 ± 1.5 ab
	Tun-Ch-MU-3	12.9 ± 2.5 abcd	1.2 ± 0.7 a	12.9 ± 3.9 abcd	1.8 ± 1.8 ab	3.4 ± 0.4 abcd	72.8 ± 6.4 abcd	24.8 ± 7.5 a	12.3 ± 15.5 ab	10.6 ± 4.3 abcdefg	0.3 ± 0.1 a	0.8 ± 0.3 a	2.2 ± 2.3 ab	2.7 ± 1.2 abc	10.9 ± 1.3 bcde	4.0 ± 0.4 ab
	Tup-Gy-AD-3-MBT	14.0 ± 2.1 abcd	1.0 ± 0.3 a	3.0 ± 2.1 a	2.7 ± 1.7 ab	3.5 ± 0.2 abcd	32.4 ± 5.0 ab	12.7 ± 4.0 a	1.2 ± 0.4 a	1.5 ± 0.6 a	0.2 ± 0.0 a	2.0 ± 0.6 ab	1.8 ± 0.8 ab	0.1 ± 0.2 ab	12.7 ± 3.0 cdef	4.7 ± 1.3 ab
	Tup-Gy-AD-6-Pi	12.1 ± 1.6 ab	1.5 ± 1.0 ab	23.2 ± 6.6 de	5.0 ± 1.8 b	4.0 ± 0.3 abcd	57.5 ± 3.7 abc	20.7 ± 8.9 a	3.9 ± 3.9 a	2.6 ± 1.5 ab	0.4 ± 0.0 a	2.0 ± 1.0 ab	1.7 ± 0.5 ab	1.5 ± 0.8 abc	9.6 ± 1.5 abcd	2.7 ± 0.8 a
	Tup-Gy-AD-6-Pr	9.7 ± 1.2 a	1.7 ± 1.4 ab	2.5 ± 0.8 a	2.6 ± 0.9 ab	2.0 ± 0.3 a	32.2 ± 12.9 ab	18.3 ± 10.9 a	1.2 ± 0.4 a	1.7 ± 0.4 ab	0.2 ± 0.1 a	2.9 ± 1.9 b	2.5 ± 0.8 ab	0.8 ± 0.5 ab	8.5 ± 2.9 abcd	2.5 ± 0.2 a
	Tup-Gy-AD-7	14.9 ± 2.8 abcd	1.0 ± 0.2 a	12.3 ± 9.6 abcd	2.2 ± 0.6 ab	4.4 ± 0.8 abcdef	40.7 ± 5.2 ab	17.6 ± 3.0 a	3.1 ± 2.9 a	4.5 ± 2.0 abc	0.3 ± 0.1 a	1.2 ± 0.4 a	2.2 ± 1.2 ab	0.0 a	13.3 ± 2.6 def	4.3 ± 0.4 ab
	Tup-Gy-AD-9	12.7 ± 0.5 abc	1.3 ± 0.2 ab	2.7 ± 1.3 a	3.1 ± 0.3 ab	3.0 ± 0.2 abc	28.7 ± 7.1 a	15.5 ± 3.3 a	1.6 ± 0.6 a	1.7 ± 0.3 ab	0.2 ± 0.0 a	1.5 ± 0.2 ab	3.0 ± 1.2 ab	1.8 ± 1.4 abc	6.8 ± 0.4 abcd	2.7 ± 0.9 a
	Tup-Gy-GA-3	15.5 ± 5.1 abcde	2.2 ± 1.3 ab	5.7 ± 3.4 abc	2.7 ± 0.4 ab	4.0 ± 1.9 abcd	45.5 ± 20.2 abc	17.2 ± 7.0 a	2.2 ± 1.7 a	8.0 ± 5.6 abcdef	0.3 ± 0.2 a	0.9 ± 0.1 a	2.7 ± 1.9 ab	2.6 ± 1.2 abc	16.4 ± 3.4 ef	4.1 ± 1.5 ab
	Tup-SJ-JM-1	12.5 ± 0.8 abc	0.7 ± 0.2 a	7.0 ± 1.5 abc	0.6 ± 0.6 a	3.2 ± 0.4 abcd	48.5 ± 4.9 abc	14.2 ± 4.0 a	8.5 ± 3.0 ab	5.4 ± 0.7 abcd	0.3 ± 0.0 a	1.3 ± 0.4 ab	1.1 ± 0.1 a	1.2 ± 0.7 abc	5.1 ± 1.1 ab	2.8 ± 0.2 ab
Departments	Lujan de Cuyo	16.8 ± 2.5 a	0.8 ± 0.2 a	17.5 ± 12.0 a	2.1 ± 1.1 a	5.0 ± 1.7 a	91.8 ± 39.0 b	17.7 ± 2.8 a	8.8 ± 2.3 a	23.8 ± 5.5 cd	0.6 ± 0.1 ab	0.6 ± 0.1 ab	2.0 ± 1.5 a	2.4 ± 1.3 a	6.5 ± 4.4 a	4.2 ± 1.2 a
	Maipu	14.8 ± 4.7 a	0.9 ± 0.0 a	4.9 ± 2.2 a	1.6 ± 0.2 a	4.1 ± 1.9 a	52.6 ± 11.2 ab	22.8 ± 4.3 a	3.2 ± 0.7 a	6.1 ± 3.6 ab	0.4 ± 0.0 a	1.6 ± 0.2 ab	1.9 ± 1.2 a	1.2 ± 1.1 a	7.6 ± 0.9 a	4.4 ± 0.2 a
	Rivadavia	19.2 ± 3.1 a	0.7 ± 0.4 a	9.5 ± 3.9 a	1.2 ± 1.1 a	4.9 ± 0.6 a	89.6 ± 30.6 ab	19.2 ± 10.5 a	34.0 ± 22.8 b	33.5 ± 10.5 d	1.4 ± 1.1 b	0.7 ± 0.2 ab	2.4 ± 2.9 a	1.6 ± 0.5 a	6.1 ± 3.0 a	4.9 ± 1.2 a
	San Carlos	17.2 ± 3.0 a	1.1 ± 0.2 a	14.8 ± 7.1 a	2.0 ± 0.6 a	5.8 ± 1.0 a	58.0 ± 7.9 ab	27.2 ± 4.6 a	6.8 ± 2.4 a	17.4 ± 2.9 bc	0.6 ± 0.1 ab	0.5 ± 0.1 a	1.6 ± 0.9 a	2.2 ± 1.4 a	8.3 ± 1.3 a	3.5 ± 0.5 a
	Tunuyan	15.7 ± 3.7 a	1.7 ± .2 a	11.5 ± 7.2 a	1.6 ± 0.3 a	4.1 ± 1.1 a	69.9 ± 20.4 ab	24.1 ± 8.3 a	10.3 ± 6.7 a	12.2 ± 6.7 abc	0.4 ± 0.2 ab	1.0 ± 0.6 ab	4.4 ± 4.0 a	2.9 ± 1.8 a	12.3 ± 5.5 a	4.4 ± 0.7 a
	Tupungato	13.1 ± 1.9 a	1.3 ± 0.5 a	8.1 ± 7.5 a	2.7 ± 1.3 a	3.5 ± 0.8 a	40.8 ± 10.4 a	16.6 ± 2.7 a	3.1 ± 2.6 a	3.6 ± 2.4 a	0.3 ± 0.1 a	1.7 ± 0.7 b	2.1 ± 0.7 a	1.1 ± 0.9 a	10.3 ± 4.0 a	3.4 ± 0.9 a
Zones	East zone	19.2 ± 3.1 a	0.7 ± 0.4 a	9.5 ± 3.9 a	1.2 ± 1.1 a	4.9 ± 0.6 a	89.6 ± 30.6 b	19.2 ± 10.5 a	34.0 ± 22.8 b	33.5 ± 10.5 b	1.4 ± 1.1 b	0.7 ± 0.2 a	2.4 ± 2.9 a	1.6 ± 0.5 a	6.1 ± 3.0 a	4.9 ± 1.2 a
	First zone	16.0 ± 3.1 a	0.9 ± 0.2 a	12.4 ± 11.0 a	1.9 ± 0.8 a	4.6 ± 1.6 a	76.1 ± 35.4 ab	19.7 ± 4.0 a	6.6 ± 3.5 a	16.7 ± 10.6 a	0.5 ± 0.2 a	1.0 ± 0.6 a	1.9 ± 1.2 a	1.9 ± 1.2 a	6.9 ± 3.2 a	4.3 ± 0.9 a
	Uco Valley	15.0 ± 3.1 a	1.3 ± 0.6 a	11.0 ± 7.5 a	2.2 ± 1.0 a	4.4 ± 1.4 a	52.3 ± 16.4 a	21.6 ± 6.6 a	5.8 ± 4.4 a	9.9 ± 7.2 a	0.4 ± 0.2 a	1.2 ± 0.7 a	2.4 ± 2.0 a	1.9 ± 1.4 a	10.1 ± 3.7 a	3.6 ± 0.8 a

Different letters within the same row indicate significant differences ($p < 0.05$) according to a Tukey HSD test.

Table S4. One-way ANOVA results for the 21 PCs (Name and PubChem CID) showing significant differences (p-value≤ 0.05) in Malbec wines from different locations of Mendoza during different

Anthocyanins

Vintage	Delphinidin 3-O-glucoside - 102515359	Cyanidin 3-O-glucoside - 197081	Petunidin 3-O-glucoside - 443651	Peonidin 3-O-glucoside - 443654	Malvidin 3-O-glucoside - 443652	Delphinidin 3-O-acetylglucoside - 15385440	Petunidin 3-O-acetylglucoside - 44256961	Peonidin 3-O-acetylglucoside - 44256847	Malvidin 3-O-acetylglucoside - 72193646	Petunidin 3-O-*p*-coumaroylglucoside - 9897848	Peonidin 3-O-*p*-coumaroylglucoside - 131752297	Malvidin 3-O-*p*-coumaroylglucoside - 71308234
2016	27.9 a	3.2 b	40.1 a	15.1 b	315.9 b	8.0 a	8.9 a	4.5 ab	28.1 c	6 b	6.5 b	16.0 c
2017	38.6 a	90.8 a	7.4 b	132.3 a	746.7 a	9.3 a	11.8 a	6.0 a	93.1 b	10.1 a	10.4 a	42.7 b
2018	23.7 a	82.1 a	0.3 b	124.8 a	798.6 a	8.4 a	12.1 a	3.6 b	116.3 a	7.1 ab	11.1 a	56.2 a

LMW-PCs

Vintage	Gallic acid - 370	OH-tyrosol - 82755	Tyrosol - 10393	(+)-Catechin - 9064	Syringic acid - 10742	(-)-Epicatechin - 72276	Astilbin - 119258	Caffeic acid - 689043	*p*-coumaric acid - 637542	Ferulic acid - 445858	*trans*-Piceid - 5281718	*trans*-resveratrol - 445154	Quercetin-3-glucoside - 5280804	Quercetin - 5280343	(-)-Gallocatechin - 9882981
2016	23 b	2.6 a	33.1 a	25.2 b	13.8 a	20.4 b	6.2 b	9.6 a	19.1 a	1.2 a	2.6 a	6.0 a	0.7 c	9.4 a	14.0 a
2017	28.3 a	2.8 a	17.8 b	31.8 a	13.2 a	32.5 b	18.9 a	7.5 a	17.2 a	0.6 b	0.9 b	5.3 a	3.9 a	11.4 a	8.0 b
2018	15.8 c	1.1 b	11.1 c	2.0 c	4.5 b	62.4 a	20.9 a	9.6 a	14.5 a	0.6 b	1.1 b	2.3 b	1.8 b	8.9 a	3.9 c

Different letters within the same row indicate significant differences ($p < 0.05$) according to a Tukey HSD test.

Table S5. Confusion matrix using PLS-DA with the 66% of data to train and 33% to test the model

Vintages	2016	2017	2018
2016	8	0	0
2017	0	8	0
2018	0	0	7

Table S6. TerraClimate data obtained from location of the parcels

Locations				Mean minimum temperature (ºC)							Mean maximum temperature (ºC)							1-Oct to 30-April		
Vintage	Zones	Departments	GIs	October	November	December	January	February	March	April	October	November	December	January	February	March	April	days with > 33 º Celsius degrees	GDD	Rainfall (mm)
2016	First Zone	Lujan de Cuyo	Agrelo	6.2	10.3	14.2	16.8	16.0	11.7	7.1	16.7	23.1	28.6	28.8	30.5	25.9	18.2	15	1755	423
			Ugarteche	5.0	9.6	14.2	15.7	15.6	10.5	6.5	20.2	24.5	29.9	29.9	30.6	26.3	19.5	NA	1795	330
		Maipu	Lunlunta	7.2	11.7	16.1	17.7	17.7	13.4	8.7	17.7	24.2	29.8	29.7	30.9	26.4	19.0	22	1952	551
	East Zone	Rivadavía	El Mirador	6.8	11.8	16.4	18.1	18.1	13.1	8.4	22.0	26.5	31.9	31.7	32.1	27.6	20.3	NA	2203	282
			La Libertad	7.9	11.4	15.5	17.9	17.4	12.5	7.5	19.3	26.3	32.0	31.1	32.3	28.1	19.7	38	2049	571
			Rivadavía	6.9	11.7	16.3	18.0	18.0	13.3	8.5	21.8	25.9	31.3	31.0	31.6	27.3	20.1	NA	2155	271
	Uco Valley	San Carlos	Altamira	6.5	9.6	13.0	15.7	15.0	11.4	6.7	15.7	23.3	29.2	29.1	30.1	25.9	17.9	17	1640	605
			El Cepillo	5.6	9.2	12.8	15.7	14.8	11.0	6.5	15.3	22.8	28.0	28.1	29.3	25.3	17.5	10	1591	769
		Tunuyan	Chacayes	5.6	9.4	13.2	15.8	14.9	11.3	6.7	16.0	22.6	27.4	27.4	28.9	24.9	17.7	7	1603	695
			Los Árboles	3.8	8.1	12.7	14.3	13.9	9.4	5.3	19.4	23.6	29.1	29.4	30.1	26.1	19.4	NA	1590	331
		Tupungato	Gualtallary	3.9	7.9	11.9	14.2	13.5	10.4	5.4	12.4	19.9	25.5	25.3	25.6	22.4	14.7	1	1225	716
			San José	6.0	9.5	14.0	16.4	15.6	11.9	7.1	15.3	22.8	29.5	28.9	30.0	25.6	17.3	13	1653	725
2017	First Zone	Lujan de Cuyo	Agrelo	7.1	10.1	13.3	16.0	15.4	11.8	8.6	22.1	26.6	31.0	33.9	31.2	27.1	21.2	38	1939	335
			Ugarteche	7.9	11.2	14.1	16.7	15.4	11.7	7.7	23.5	28.2	31.2	33.4	31.0	26.9	21.8	NA	2129	230
		Maipu	Lunlunta	8.9	11.8	14.7	18.0	16.7	13.6	10.3	22.7	27.5	30.4	33.1	30.9	27.1	21.2	37	2185	279
	East Zone	Rivadavía	El Mirador	9.6	12.9	15.7	17.3	16.1	12.6	9.0	25.5	30.5	33.7	36.7	34.3	30.4	24.0	85	2491	319
			La Libertad	9.6	12.9	15.3	17.2	17.2	14.5	10.4	25.5	30.5	33.9	37.6	33.8	29.0	22.9	80	2496	183
			Rivadavía	8.5	10.8	14.1	17.4	16.5	13.2	10.1	26.0	30.9	34.3	38.5	35.4	31.1	24.7	96	2459	287
	Uco Valley	San Carlos	Altamira	6.5	9.1	11.9	14.5	14.2	10.2	7.4	21.4	26.3	29.7	33.4	30.1	26.7	20.2	34	1846	478
			El Cepillo	4.8	7.7	10.7	13.3	14.3	12.1	8.5	21.0	25.6	29.5	32.6	29.3	25.7	19.7	27	1797	541
		Tunuyan	Chacayes	6.3	8.5	11.8	14.0	14.1	9.8	7.1	21.0	25.3	28.5	31.6	28.7	25.9	19.9	20	1806	379
			Los Árboles	6.4	9.4	12.3	14.6	13.3	9.6	6.2	22.1	27.3	30.5	32.8	30.6	26.6	21.6	NA	1866	250
		Tupungato	Gualtallary	4.1	6.8	9.6	12.3	13.3	11.2	7.9	18.1	23.1	26.4	30.0	26.8	23.7	17.3	7	1438	368
			San José	7.0	10.3	12.9	15.7	15.1	11.8	8.0	21.6	27.1	31.2	34.3	30.8	26.8	20.4	42	1952	408
2018	First Zone	Lujan de Cuyo	Agrelo	5.4	10.2	14.3	15.5	14.9	8.7	8.1	24.6	28.1	30.0	32.0	31.5	28.6	24.6	39	2028	162
			Ugarteche	7.3	11.5	14.7	15.1	14.9	10.0	9.2	24.6	28.4	31.1	31.5	31.1	28.0	23.8	NA	2138	140
		Maipu	Lunlunta	8.2	12.1	15.8	16.7	16.2	11.6	10.3	24.1	27.8	29.9	30.6	30.6	28.2	25.0	26	2184	195
	East Zone	Rivadavía	El Mirador	5.9	10.3	15.2	15.9	15.4	7.6	8.6	26.9	31.0	33.4	34.6	33.6	31.8	28.0	90	2433	240
			La Libertad	6.9	11.2	15.5	16.2	15.5	9.2	9.3	25.8	30.7	32.9	33.9	33.0	30.2	26.1	70	2321	124
			Rivadavía	7.2	11.2	15.4	16.2	15.3	9.4	9.3	27.6	30.7	33.6	35.2	34.0	31.4	27.4	92	2389	130
	Uco Valley	San Carlos	Altamira	5.1	9.0	13.2	14.0	13.2	7.9	7.1	23.5	27.5	29.7	31.1	30.8	28.4	24.2	33	1882	116
			El Cepillo	4.4	8.4	12.8	13.8	13.1	7.4	7.0	22.8	26.4	28.6	29.9	29.0	27.0	23.3	9	1794	202
		Tunuyan	Chacayes	4.2	8.7	13.2	14.1	13.1	7.0	7.1	23.1	26.5	28.6	29.6	29.3	27.1	23.7	14	1850	73
			Los Árboles	5.7	9.6	13.0	13.1	12.9	8.0	7.6	23.2	27.3	30.3	31.1	30.7	27.8	23.6	NA	1874	146
		Tupungato	Gualtallary	4.3	8.2	12.2	13.1	12.4	7.5	6.2	20.4	24.2	26.2	26.7	26.3	24.7	21.0	2	1432	216
			San José	6.2	10.3	14.4	15.1	14.6	9.3	8.4	23.7	27.8	30.3	31.3	30.8	28.2	23.7	34	1965	162

Table S7. Chemical characteristics [Mean ± SD] of must and wines from different locations of Mendoza.

Locations				Must analysis					Wine Analysis						
Zones	Departments	IGs	Parcels	Vintage	º Brix	pH	Total acidity (g L -1 tartaric acid)	Number of replicates of microvinifications	Alcohol level % v/v	Total acidity (g L -1 tartaric acid)	pH	Volatile acidity (g L -1 acetic acid)	Residual sugar (g L -1)	Color intensity (Absorbance at 420 nm + Absorbance at 520 nm)	Hue (Absorbance at 420 nm/Absorbance at 520 nm)
First Zone	Lujan de Cuyo	Agrelo	Luj-Agr-LP-1-80	2016	23.0	3.59	7.7	3	13.4 ± 0.1	6.3 ± 0.1	3.67 ± 0.14	0.47 ± 0.11	0.1 ± 0.1	11.94 ± 1.83	0.61 ± 0.8
				2017	24.0	3.59	5.8	2	14.2 ± 0.1	4.9 ± 0.1	3.72 ± 0.02	0.53 ± 0.10	3.0 ± 0.1	9.42 ± 0.78	0.47 ± 0.01
				2018	24.6	3.61	5.1	3	14.1 ± 0.2	6.3 ± 0.4	3.53 ± 0.03	0.79 ± 0.23	2.9 ± 0.1	16.24 ± 1.17	0.58 ± 0.01
		Ugarteche	Luj-Ug-FO-1	2016	23.4	3.46	7.7	3	13.8 ± 0.5	4.9 ± 0.1	3.58 ± 0.01	0.46 ± 0.04	0.5 ± 0.2	13.71 ± 1.06	0.55 ± 0.01
				2017	24.6	3.83	4.3	3	14.7 ± 0.3	4.8 ± 0.4	3.73 ± 0.04	0.46 ± 0.04	1.1 ± 0.6	10.86 ± 0.31	0.47 ± 0.01
				2018	26.0	3.69	4.5	3	14.6 ± 0.3	5.7 ± 0.4	3.55 ± 0.01	0.75 ± 0.10	2.0 ± 1.0	9.52 ± 0.79	0.66 ± 0.01
			Luj-Ug-ZA-1	2016	21.0	3.62	7.8	3	11.5 ± 0.2	4.9 ± 0.1	3.48 ± 0.03	0.37 ± 0.05	0.4 ± 0.1	8.65 ± 0.85	0.56 ± 0.01
				2017	21.0	3.97	3.7	2	12.3 ± 0.1	5.0 ± 0.6	3.77 ± 0.05	0.53 ± 0.23	2.2 ± 0.3	10.32 ± 0.76	0.47 ± 0.01
				2018	23.6	3.68	4.5	3	12.9 ± 0.1	6.6 ± 0.5	3.58 ± 0.01	0.97 ± 0.03	3.9 ± 0.9	9.49 ± 0.70	0.66 ± 0.00
	Maipu	Lunlunta	Mai-Lu-AG-18	2016	23.0	3.72	5.9	3	12.7 ± 0.4	5.4 ± 0.4	3.63 ± 0.02	0.63 ± 0.04	0.2 ± 0.1	12.07 ± 2.01	0.56 ± 0.03
				2017	24.0	3.38	6.3	3	14.4 ± 0.1	4.8 ± 0.2	3.67 ± 0.09	0.49 ± 0.12	2.9 ± 0.1	10.05 ± 2.41	0.47 ± 0.03
				2018	24.2	3.85	3.9	3	14.2 ± 0.4	5.1 ± 0.2	3.57 ± 0.02	0.66 ± 0.23	2.1 ± 0.1	11.84 ± 0.96	0.60 ± 0.02
			Mai-Lu-AG-20	2016	24.6	3.59	5.7	3	14.3 ± 0.3	5.2 ± 0.4	3.55 ± 0.10	0.46 ± 0.06	2.3 ± 1.1	18.46 ± 1.31	0.54 ± 0.01
				2017	23.6	3.75	5.2	3	14.4 ± 0.2	4.9 ± 0.1	3.69 ± 0.06	0.50 ± 0.06	2.9 ± 0.2	8.47 ± 0.98	0.49 ± 0.02
				2018	25.4	3.81	3.8	3	15.1 ± 0.3	5.4 ± 0.2	3.53 ± 0.06	0.61 ± 0.05	2.0 ± 0.5	10.22 ± 0.39	0.61 ± 0.01
East Zone	Rivadavia	El Mirador	Riv-Mir-LV-4	2016	22.6	3.80	5.8	3	12.7 ± 0.5	4.7 ± 0.3	3.67 ± 0.02	0.82 ± 0.17	0.6 ± 0.1	7.91 ± 0.56	0.66 ± 0.02
				2017	21.6	3.75	4.1	3	13.1 ± 0.1	4.8 ± 0.2	3.80 ± 0.04	0.50 ± 0.06	2.3 ± 0.5	8.00 ± 0.25	0.52 ± 0.01
				2018	24.4	3.95	4.0	3	14.9 ± 0.3	4.9 ± 0.3	3.63 ± 0.08	0.54 ± 0.03	2.4 ± 0.6	9.30 ± 0.68	0.66 ± 0.02
		La Libertad	Riv-Lib-LL-1	2016	24.6	3.79	5.7	3	14.8 ± 0.4	5.2 ± 0.5	3.88 ± 0.15	0.82 ±0.08	3.9 ± 2.4	9.49 ± 0.78	0.72 ± 0.02
				2017	24.0	3.38	6.3	3	13.4 ± 0.4	5.7 ± 0.6	3.76 ± 0.02	0.67 ± 0.09	1.2 ± 0.5	7.58 ± 0.63	0.52 ± 0.01
				2018	24.4	3.83	4.5	3	13.7 ± 0.1	5.7 ± 0.1	3.61 ± 0.05	0.91 ± 0.09	2.6 ± 0.7	6.91 ± 0.21	0.72 ± 0.01
		Rivadavia	Riv-Riv-LA-1	2016	21.6	3.79	5.7	3	12.7 ± 0.4	6.8 ± 0.3	3.73 ± 0.02	0.79 ± 0.11	1.7 ± 1.5	6.95 ± 0.07	0.71 ± 0.01
				2017	22.6	3.80	5.4	3	13.1 ± 0.2	5.1 ± 0.5	3.72 ± 0.11	0.75 ± 0.12	2.2 ± 0.3	4.23 ± 0.25	0.62 ± 0.03
				2018	24.2	3.91	4.2	3	13.4 ± 0.1	6.0 ± 0.2	3.61 ± 0.03	1.16 ± 0.30	1.5 ± 0.1	5.93 ± 0.37	0.76 ± 0.01
Uco Valley	San Carlos	Altamira	San-Alt-NI-1-Pi	2016	22.6	3.64	6.9	2	13.7 ± 0.1	5.3 ± 0.3	3.65 ± 0.04	0.45 ± 0.12	0.4 ± 0.5	12.80 ± 0.24	0.53 ± 0.01
				2017	25.0	3.58	4.3	3	14.7 ± 0.3	5.1 ± 0.2	3.70 ± 0.02	0.50 ± 0.02	3.1 ± 0.2	14.00 ± 0.91	0.50 ± 0.01
				2018	23.2	3.46	4.9	3	14.3 ± 0.0	5.7 ± 0.1	3.52 ± 0.20	0.60 ± 0.08	2.5 ± 0.1	18.06 ± 0.77	0.57 ± 0.01
			San-Alt-NI-1-Pr	2016	22.6	3.57	7.1	3	12.7 ± 0.1	4.9 ± 0.1	3.56 ± 0.02	0.61 ± 0.03	0.5 ± 0.4	6.37 ± 0.10	0.62 ± 0.02
				2017	25.0	3.63	5.2	3	15.1 ± 0.2	5.3 ± 0.1	3.65 ± 0.06	0.53 ± 0.06	1.9 ± 0.4	11.56 ± 0.76	4.19 ± 0.08
				2018	24.8	3.44	5.3	3	13.8 ± 0.1	5.2 ± 0.2	3.58 ± 0.05	0.55 ± 0.07	2.2 ± 0.2	11.84± 0.82	0.60 ± 0.01
			San-Alt-NI-2-Pr	2016	22.0	3.62	7.6	3	12.7 ± 0.2	4.5 ± 0.1	3.69 ± 0.01	0.36 ± 0.06	0.2 ± 0.1	8.98 ± 0.69	0.61 ± 0.01
				2017	24.6	3.71	4.6	3	14.9 ± 0.2	4.7 ± 0.1	3.80 ± 0.08	0.58 ± 0.06	1.8 ± 0.2	16.30 ± 2.37	0.48 ± 0.03
				2018	24.0	3.58	4.5	3	14.2 ± 0.0	5.0 ± 0.1	3.62 ± 0.03	0.47 ± 0.01	2.1 ± 0.2	16.20 ± 0.56	0.58 ± 0.01
		El Cepillo	San-Cep-EB-3	2016	23.6	3.60	6.9	3	13.5 ± 0.3	6.2 ± 0.6	3.51 ± 0.12	0.55 ± 0.07	0.5 ± 0.8	18.38 ± 1.31	0.54 ± 0.01
				2017	23.4	2.55	4.8	3	14.0 ± 0.3	5.3 ± 0.3	3.67 ± 0.01	0.52 ± 0.16	1.3 ± 0.2	19.29 ± 0.98	0.44 ± 0.01
				2018	24.4	3.53	4.3	3	14.6 ± 0.0	5.4 ± 0.1	3.53 ± 0.03	0.57 ± 0.04	2.7 ± 0.1	14.44 ± 0.22	0.59 ± 0.01
			San-Cep-EC-5	2016	22.6	3.60	6.7	3	12.9 ± 0.1	5.1 ± 0.4	3.72 ± 0.04	0.30 ± 0.04	0.3 ± 0.1	12.43 ± 0.91	0.56 ± 0.01
				2017	25.0	3.66	5.5	2	15.1 ± 0.4	5.4 ± 0.3	3.71 ± 0.10	0.46 ± 0.09	2.1 ± 0.9	15.05 ± 0.92	0.51 ± 0.01
				2018	24.2	3.60	5.3	3	14.2 ± 0.1	5.8 ± 0.2	3.59 ± 0.05	0.59 ± 0.08	2.0 ± 0.5	15.66 ± 0.63	0.60 ± 0.01
	Tunuyan	Chacayes	Tun-Ch-MU-3	2016	23.0	3.59	6.2	3	13.3 ± 0.1	4.7 ± 0.1	3.66 ± 0.01	0.22 ± 0.04	0.3 ± 0.1	13.67 ± 0.54	0.53 ± 0.01
				2017	25.0	3.68	4.6	2	14.9 ± 0.4	5.0 ± 0.1	3.69 ± 0.02	0.57 ± 0.02	1.4 ± 0.5	16.56 ± 2.22	0.47 ± 0.01
				2018	25.6	3.70	4.8	3	14.7 ± 0.4	6.4 ± 0.2	3.59 ± 0.09	0.86 ± 0.02	8.3 ± 4.6	14.29 ± 0.86	0.57 ± 0.02
		Los Arboles	Tun-Al-BV-5	2016	22.6	3.59	6.9	3	13.5 ± 0.2	5.3 ± 0.2	3.58 ± 0.03	0.34 ± 0.09	1.6 ± 1.6	17.17 ± 0.65	0.51 ± 0.01
				2017	24.4	3.67	4.2	3	15.4 ± 0.8	5.2 ± 0.2	3.67 ± 0.03	0.55 ± 0.15	3.1 ± 0.5	24.41 ± 1.00	0.45 ± 0.01
				2018	25.0	3.50	5.9	3	15.1 ± 0.3	6.1 ± 0.1	3.56 ± 0.01	0.57 ± 0.01	2.4 ± 0.1	20.29 ± 0.63	0.60 ± 0.01
			Tun-Al-CC-1	2016	21.0	3.60	7.4	3	12.6 ± 0.2	4.9 ± 0.1	3.56 ± 0.01	0.25 ± 0.04	0.2 ± 0.1	11.51 ± 0.50	0.54 ± 0.2
				2017	24.0	3.75	4.6	3	14.9 ± 0.3	5.6 ± 0.6	3.65 ± 0.07	0.49 ± 0.08	1.4 ± 0.3	14.86 ± 0.81	0.48 ± 0.01
				2018	26.0	3.56	5.3	3	15.5 ± 0.0	6.5 ± 0.4	3.56 ± 0.10	0.60 ± 0.03	4.7 ± 0.8	22.09 ± 0.79	0.59 ± 0.01
	Tupungato	Gualtallary	Tup-Gy-AD-3-MBT	2016	24.0	3.45	5.7	3	13.6 ± 0.1	6.2 ± 0.1	3.29 ± 0.02	0.24 ± 0.02	0.6 ± 0.1	18.75 ± 1.95	0.45 ± 0.02
				2017	24.0	3.45	5.7	3	14.1 ± 0.3	5.7 ± 0.1	3.65 ± 0.03	0.31 ± 0.04	1.6 ± 0.8	26.58 ± 1.82	0.40 ± 0.01
				2018	25.0	3.62	6.0	3	14.7 ± 0.2	6.2 ± 0.3	3.45 ± 0.04	0.30 ± 0.03	1.8 ± 0.2	17.52 ± 1.14	0.57 ± 0.01
			Tup-Gy-AD-6-Pi	2016	22.6	3.38	8.0	3	13.1 ± 0.1	5.7 ± 0.2	3.40 ± 0.02	0.20 ± 0.01	0.4 ± 0.1	17.18 ± 0.80	0.46 ± 0.01
				2017	24.0	3.38	6.3	3	14.7 ± 0.4	5.6 ± 0.2	3.67 ± 0.08	0.40 ± 0.23	1.5 ± 0.3	26.59 ± 3.53	0.42 ± 0.01
				2018	24.0	3.39	6.6	3	13.9 ± 0.1	5.8 ± 0.1	3.52 ± 0.01	0.45 ± 0.07	1.5 ± 0.1	14.00 ± 1.32	0.57 ± 0.01
			Tup-Gy-AD-6-Pr	2016	23.0	3.34	7.6	3	13.4 ± 0.1	4.0 ± 0.1	3.55 ± 0.02	0.42 ± 0.02	0.2 ± 0.1	18.68 ± 1.59	0.46 ± 0.01
				2017	25.0	3.41	6.2	3	15.5 ± 0.2	6.6 ± 0.5	3.43 ± 0.06	0.29 ± 0.09	3.6 ± 1.0	33.18 ± 2.65	0.47 ± 0.13
				2018	25.8	3.34	6.0	3	15.1 ± 0.4	6.4 ± 0.2	3.48 ± 0.04	0.40 ± 0.06	4.2 ± 1.4	22.16 ± 3.47	0.57 ± 0.01
			Tup-Gy-AD-7	2016	23.0	3.57	8.6	2	13.9 ± 0.4	6.6 ± 0.2	3.29 ± 0.02	0.31 ± 0.03	2.5 ± 2.7	18.70 ± 1.81	0.47 ± 0.01
				2017	25.0	3.51	6.5	3	14.9 ± 0.4	6.1 ± 0.1	3.51 ± 0.04	0.40 ± 0.06	3.4 ± 0.4	30.92 ± 0.17	0.56 ± 0.05
				2018	23.6	3.30	7.5	3	13.5 ± 0.4	5.7 ± 0.1	3.50 ± 0.03	0.48 ± 0.10	1.7 ± 0.1	14.22 ± 1.94	0.57 ± 0.01
			Tup-Gy-AD-9	2016	23.0	3.37	7.8	3	13.4 ± 0.1	7.1 ± 0.1	3.38 ± 0.01	0.13 ± 0.03	0.5 ± 0.3	18.44 ± 0.58	0.45 ± 0.01
				2017	25.0	5.52	6.2	3	14.8 ± 0.2	5.5 ± 0.1	3.72 ± 0.09	0.45 ± 0.08	3.0 ± 0.2	23.20 ± 0.91	0.43 ± 0.01
				2018	25.4	3.41	6.6	3	14.8 ± 0.1	6.1 ± 0.1	3.49 ± 0.04	0.44 ± 0.05	2.0 ± 0.5	16.88 ± 0.74	0.57 ± 0.01
			Tup-Gy-GA-3	2016	21.0	3.34	9.5	3	11.8 ± 0.2	6.5 ± 0.1	3.61 ± 0.03	0.20 ± 0.05	0.2 ± 0.1	13.63 ± 0.71	0.49 ± 0.01
				2017	24.0	3.51	6.0	3	13.8 ± 0.4	5.0 ± 0.2	3.76 ± 0.07	0.40 ± 0.08	2.8 ± 0.5	21.91 ± 0.67	0.40 ± 0.02
				2018	23.6	3.28	7.9	3	14.8 ± 0.1	6.6 ± 0.1	3.51 ± 0.02	0.39 ± 0.03	2.6 ± 0.3	18.05 ± 2.73	0.57 ± 0.01
		San José	Tup-SJ-JM-1	2016	22.6	3.55	6.5	3	13.3 ± 0.1	5.4 ± 0.4	3.48 ± 0.07	0.28 ± 0.07	0.3 ± 0.1	12.97 ± 0.37	0.51 ± 0.01
				2017	23.0	3.48	5.1	3	13.6 ± 0.1	4.8 ± 0.1	3.65 ± 0.01	0.47 ± 0.05	2.4 ± 0.1	13.98 ± 1.13	0.42 ± 0.01
				2018	25.4	3.62	5.1	3	14.6 ± 0.1	5.4 ± 0.1	3.54 ± 0.01	0.55 ± 0.02	2.4 ± 0.3	14.66 ± 0.27	0.59 ± 0.01
